# Temporizin-1 Meets the Membranes: Probing Membrane Inser-Tion and Disruption Mechanisms

**DOI:** 10.3390/antibiotics14090913

**Published:** 2025-09-10

**Authors:** Rosa Bellavita, Sara Palladino, Karyne Rangel, Guilherme Curty Lechuga, Lorenzo Emiliano Imbò, Lucia Falcigno, Gabriella D’Auria, Leonardo da Silva Lara, Mirian Cláudia de Souza Pereira, Salvatore Giovanni De-Simone, Stefania Galdiero, Annarita Falanga

**Affiliations:** 1 Department of Pharmacy, School of Medicine, University of Naples Federico II, Via Domenico Montesano 49, 80131 Napoli, Italy; rosa.bellavita@unina.it (R.B.); sara.palladino@unina.it (S.P.); falcigno@unina.it (L.F.); gabriella.dauria@unina.it (G.D.); 2 Center for Technological Development in Health (CDTS), National Institute of Science and Technology for Innovation in Neglected Population Diseases (INCT-IDPN), Oswaldo Cruz Foundation (FIOCRUZ), Rio de Janeiro 21040-900, Brazil; karyne.rangelk@gmail.com (K.R.); guilherme.curty@fiocruz.br (G.C.L.); salvatore.simone@fiocruz.br (S.G.D.-S.); 3 Laboratory of Epidemiology and Molecular Systematics (LESM), Oswaldo Cruz Institute, Oswaldo Cruz Foundation (FIOCRUZ), Rio de Janeiro 21040-900, Brazil; 4 Cellular Ultrastructure Laboratory, Oswaldo Cruz Institute, Oswaldo Cruz Foundation (FIOCRUZ), Rio de Janeiro 21040-900, Brazil; leonardosilva.lara@hotmail.com (L.d.S.L.); mirian@ioc.fiocruz.br (M.C.d.S.P.); 5 Department of Agricultural Sciences, University of Naples Federico II, Via Università 100, 80055 Portici, Italy; lorenzoemiliano.imbo@unina.it

**Keywords:** antimicrobial peptides, physical–chemical properties, membrane interaction, *Trypanosoma cruzi*, Temporizin-1 NMR structure

## Abstract

**Background/Objectives:** Temporizin-1, a hybrid antimicrobial peptide derived from the combination of Temporin A, Gramicidin peptide, and a poly-leu sequence, has strong trypanocide activity against *Trypanosoma cruzi* and moderate cytotoxicity towards mammalian cells. In this study, we investigated the mode of action of the peptide upon interaction with protozoan and eukaryotic membranes. **Methods:** To this end, we conducted a series of biophysical assays using liposomes as biomimetic models, along with fluorescence-based experiments such as lipid mixing, membrane leakage, and assays involving Thioflavin and Laurdan. **Results:** Temporizin-1 displayed potent membranolytic activity on protozoan and eukaryotic membranes, causing significant membrane fusion and leakage with consequent pore formation. In addition, we also performed structural studies on liposome interaction, where we observed a helical structure that is conserved during membrane interaction. The NMR study confirms all the data obtained, providing both the structure of free Temporizin-1 in solution and the way it interacts with micelles. Moreover, Temporizin-1 demonstrated high selectivity against intracellular forms of *T. cruzi* and exhibited an additive effect when combined with benznidazole, highlighting its promising therapeutic activity. **Conclusions:** In conclusion, elucidating the mechanism of action of Temporizin-1 is essential for optimizing its structure and improving target selectivity, and driving the rational design of next-generation antimicrobial peptides by applying chemical strategies and delivery system’s conjugation.

## 1. Introduction

Chagas disease, caused by the hemoflagellate protozoan *Trypanosoma cruzi*, remains one of the most neglected tropical diseases in the world, predominantly affecting low-income populations [1,2]. It is endemic in 21 countries across the Americas, with the highest prevalence in rural areas of Bolivia, Argentina, Brazil, Colombia, and Mexico [3]. However, due to human migration, cases have been reported in non-endemic countries such as the United States, Spain, and Australia, where transmission occurs mainly through congenital or transfusional routes [4]. As a matter of fact, this disease exhibits complex epidemiology, with multiple transmission routes that include not only the traditional triatomine vector but also congenital transmission, blood transfusion, and oral transmission, significantly complicating its control [4,5,6]. Despite global efforts in disease control and improved diagnostics, *T. cruzi* poses a serious public health challenge, with millions of people infected and at risk of developing chronic complications [7]. The World Health Organization (WHO) estimates that approximately 6 to 7 million individuals are infected, with around 70 million living in at-risk areas [8].

The disease progresses through two distinct phases: acute and chronic. The acute phase is often asymptomatic or presents with fever, lymphadenopathy, and, in severe cases, myocarditis or meningoencephalitis [9]. The chronic phase may remain indeterminate or progress to Chagas cardiomyopathy (30–40% of cases) [10] and megacolon/megaesophagus (10%) [11]. Current treatment relies on two main drugs, benznidazole and nifurtimox, which exhibit variable efficacy (60–80% in the acute phase, <20% in the chronic phase) and severe adverse effects such as allergies, neuropathies, and hepatotoxicity, often leading to treatment discontinuation [12]. Additionally, resistance of *T. cruzi* to these compounds has been reported, underscoring the urgent need for new therapeutic alternatives [13].

In this context, antimicrobial peptides (AMPs) emerge as promising therapeutic candidates due to their broad-spectrum activity against many pathogens, including bacteria, fungi, viruses, and protozoa [14,15]. These molecules, essential components of innate immunity, act through multifactorial mechanisms, reducing the likelihood of resistance development [16,17,18]. Recent studies have demonstrated the efficacy of AMPs against trypanosomatids, including *T. cruzi*, highlighting their potential as a therapeutic alternative [19,20,21]. AMPs combat *T. cruzi* through multifaceted mechanisms, where their positive charge allows binding to anionic phospholipids in the parasite’s membrane, inducing pore formation and cell lysis [22]. Additionally, they modulate the host immune response stimulating the production of pro-inflammatory cytokines (such as IL-12 and TNF-α), which enhance parasite clearance [23]. Several families of AMPs have demonstrated trypanocidal activity, including defensins, cathelicidins, cecropins, and temporins, each with specific mechanisms of action against *T. cruzi* [22]. Defensins, particularly human β-defensin 1 (HBD-1) and α-defensin 1 (HNP-1), have shown the ability to significantly reduce the infectivity of the parasite’s trypomastigote forms [24]. Cathelicidins, such as human-derived LL-37, act by disrupting the parasite’s mitochondrial membrane, leading to its death [22]. Cecropins, originally isolated from insects, exhibit potent trypanocidal activity at micromolar concentrations, with cecropin A showing particular efficacy against intracellular forms of the parasite [22].

Among AMPs, two temporin-derived molecules, Temporizin (FLPLWLWLWLWLWKLK) and its synthetic analog Temporizin-1 (FLPLWLWLWRKLK), deserve particular attention for their trypanocidal potential. Temporizin is a synthetic hybrid peptide composed of three distinct regions: the N-terminal segment of Temporin A, from the skin of the amphibian *Rana temporaria* [25]; the pore-forming domain of Gramicidin; and a C-terminal tail featuring alternating leucine and lysine residues. Although AMPs generally offer significant therapeutic potential, some can also penetrate mammalian plasma membranes, leading to undesirable cytotoxic effects. Temporizin demonstrated an EC_50_ of 116.9 μg/mL against mammalian cells, while exhibiting potent activity against *Trypanosoma* spp. at concentrations approximately 100-fold lower, indicating a favorable therapeutic index and selective toxicity toward the parasite [25]. To further improve its safety profile, Temporizin-1, characterized by a truncated pore-forming domain, was engineered to reduce its interaction with lipid bilayers and consequently cytotoxicity against mammalian cells while maintaining antiparasitic efficacy [25].

To better understand the enhanced activity of Temporizin-1 against *Trypanosoma* spp. alongside its reduced cytotoxicity toward mammalian cells, it is essential to investigate its mechanism of action using membrane-mimicking systems. Model membranes that replicate the distinct lipid compositions of protozoan and mammalian cells provide a valuable platform for elucidating these interactions at the molecular level and further optimizing the AMP’s structure for therapeutic applications, enhancing selectivity, minimizing off-target effects, and informing the rational design of next-generation therapeutics. Moreover, the significant antiparasitic activity of Temporizin-1 was also confirmed against the trypomastigote and intracellular amastigote forms, and its additive effect was observed when administered in combination with the traditional benznidazole.

## 2. Results

### 2.1. Temporizin-1 and Physicochemical Properties

**Figure 1:**
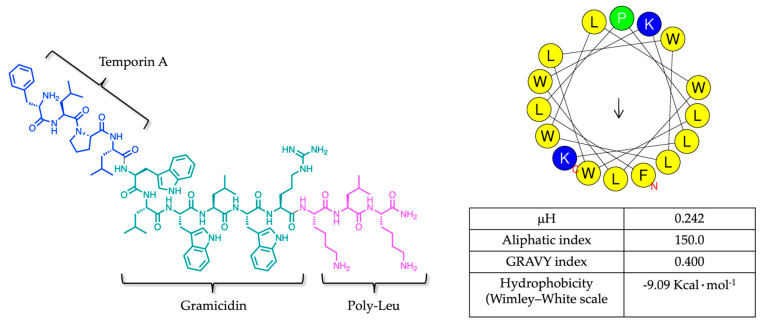
Representation of Temporizin-1’s sequence and helical wheel projection obtained by HeliQuest website. The table reports its physicochemical properties calculated by HeliQuest, ExPASy, and MPEx software.

Temporizin-1 is a synthetic hybrid peptide designed by Souza et al. derived from a combination of some fragments of Temporin A, Gramicidin, and a poly-leu sequence as shown in Figure 1 [25]. The peptide is rich in hydrophobic amino acids, particularly due to the repeated WL motif, which likely facilitates its interaction with lipid bilayers and insertion into the membrane. Specifically, Temporizin-1 contains three positively charged residues, such as lysine and arginine amino acids (R10, K11, K13), and nine hydrophobic residues, including one phenylalanine (F1), three tryptophan residues (W5, W7, W9), and five leucine residues (L2, L4, L6, L8, L12). In Figure 1, the helical wheel projection (obtained from the HeliQuest website) shows the hydrophobic residues located on the hydrophobic face, colored in yellow, and the hydrophilic residues on the hydrophilic face of the helix, colored blue.

The physicochemical properties of Temporizin-1, including amphipathicity and hydrophobicity, were analyzed through the calculation of its hydrophobic moment by *HeliQuest*, aliphatic index and GRAVY (Grand Average of Hydropathy) index by *ExPASy*, and Wimley–White hydrophobicity scale obtained from Membrane Protein Explore (MPEx) software (Version 3.3.1) (Figure 1). The hydrophobic moment (μH) quantifies both the amphipathicity and hydrophobicity of the non-polar face of an amphipathic α-helix, influencing the orientation and manner in which the peptide interacts with the lipid bilayer [26]. The μH value of 0.242 indicates a low amphipathicity and high hydrophobicity that could drive deeper penetration inside the lipid bilayer [27,28]. The peptide’s hydrophobic character is further supported by an aliphatic index of 150.0, determined as the relative volume occupied by aliphatic side chains, and the GRAVY index of 0.400. The GRAVY index is calculated as the sum of hydropathy values of all amino acids, and the positive value supports the hydrophobic nature of Temporizin-1 [29].

Additionally, we calculated hydrophobicity through the Wimley–White scale, a biochemical tool that measures the free energy change associated with transferring a peptide from an aqueous environment to a hydrophobic medium, such as octanol, which mimics a membrane [30,31]. The interfacial scale (IF), measuring a residue’s free energy of transfer within a peptide from water to a phosphocholine bilayer interface, give a negative Gibbs energy of −9.09 Kcal∙mol^−1^ indicating a favorable membrane insertion of Temporizin-1.

### 2.2. Temporizin-1 Induces the Fusion Membrane and Leakage

**Figure 2:**
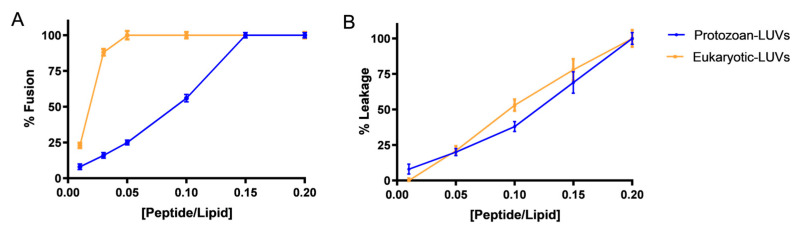
Panel (**A**): Ability of Temporizin-1 to induce membrane fusion in the presence of 100 μM LUVs containing 0.6% NBD and 0.6% Rho. The increase in fluorescence was measured after the addition of peptide aliquots. Panel (**B**): The leakage percentage of protozoan and eukaryotic LUVs after the peptide addition. The calcein release was monitored recording the fluorescence increase at 535 nm after the peptide addition. Data points are the mean ± SD of three different experiments.

To elucidate the specific mechanism of action, we employed large unilamellar vesicles (LUVs) designed to mimic the lipid composition of both eukaryotic, [phosphatidylcholine–cholesterol—PC:Chol, 70:30 mol%] and protozoan membranes [phosphatidylcholine–phosphatidylethanolamine–phosphatidylinositol–sphingomyelin–phosphatidylserine–ergosterol—PC:PE:PI:SM:PS:Erg, 35:30:15:5:10:5 mol%] [32]. Firstly, we investigated the fusogenic activity of Temporizin-1 by performing lipid mixing assays and using LUVs labeled with a FRET pair consisting of Rhodamine (Rho) and 12-(N-methyl-N-(7-nitrobenz-2-oxa-1,3-diazol-4-yl)) (NBD)–phosphatidylethanolamine (PE) as acceptor and donor probes, respectively. Labeled and unlabeled LUVs were mixed at the ratio 1:4, and the extent of lipid mixing was monitored as a function of peptide/lipid (P/L) molar ratio. Temporizin-1 induced membrane fusion both in the presence of LUVs mimicking eukaryotic and protozoan membranes, but with a different trend (Figure 2A). Specifically, the peptide displayed significant ability to induce fusion of eukaryotic LUVs already at the P/L of 0.05; in contrast, Temporizin-1 induced the complete fusion of protozoan-mimetic LUVs at a higher P/L of 0.15.

Additionally, we assessed the ability of the peptide to form pores, evaluating the release of calcein encapsulated inside the LUVs, upon the addition of increasing peptide concentrations [33]. Figure 2B shows that Temporizin-1 is able to induce pore formation both in LUVs mimicking protozoan and eukaryotic membranes at all P/L ratios. Specifically, in both conditions, the peptide caused 100% liposome disruption at the highest P/L ratio of 0.2.

### 2.3. Temporizin-1 Embeds Itself Within the Lipid Bilayer

We also investigated the capacity of Temporizin-1 to directly interact with membranes, analyzing the quenching of the tryptophans (Trp) present in the peptide by acrylamide in water and in the presence of protozoan and eukaryotic LUVs. Acrylamide is water-soluble and highly efficient in quenching Trp, and it is unable to penetrate the hydrophobic core of the lipid bilayer. Thus, the more deeply Trp residues are buried, the less efficiently they are quenched by acrylamide.

**Figure 3:**
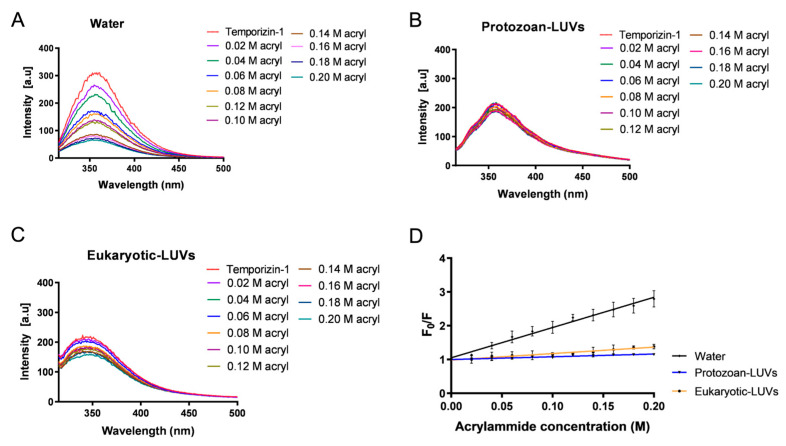
Tryptophan fluorescence spectra of Temporizin-1 during quenching with increasing concentrations of acrylamide: in water (panel (**A**)), in Protozoan LUVs (panel (**B**)), and in eukaryotic LUVs (panel (**C**)). Panel (**D**) shows Stern–Volmer plots of acrylamide quenching of Temporizin-1 in water and in LUVs.

Fluorescence measurements in water showed a concentration-dependent quenching of Trp residues upon the addition of acrylamide to the peptide solution (Figure 3A), indicating that the residues are accessible and susceptible to quenching. In contrast, a different behavior can be observed in the presence of both liposome compositions, where the tryptophan residues appeared less accessible and thus more buried within the lipid bilayer (Figure 3B,C).

The degree of Trp accessibility was quantitatively assessed by calculating Stern–Volmer quenching constants (K_sv_) through linear regression of the fluorescence data (Figure 3D). This plot clearly indicates that quenching is stronger in water, K_sv_ = 8.9 ± 0.2, while in liposomes the K_sv_ values are lower, 0.81 ± 0.05 and 1.9 ± 0.1 for protozoan and eukaryotic LUVs, respectively, suggesting a deeper insertion in LUVs of tryptophan residues.

**Figure 4:**
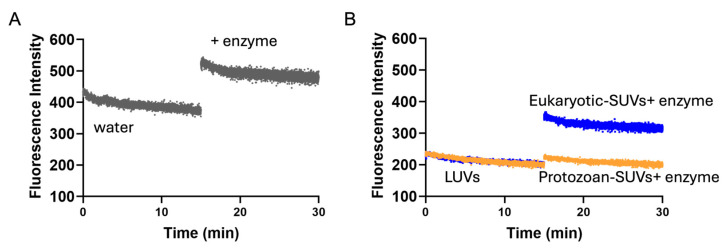
Proteolytic digestion of Temporizin-1 in water (**A**) and in protozoan and eukaryotic LUVs (**B**). The fluorescence emission spectra of the tryptophan residues were obtained at 354 nm with excitation set at 295 nm before and after the addition of chymotrypsin enzyme.

To confirm the insertion of Temporizin-1 into liposomes and to verify that its Trp residues remain shielded from the aqueous phase in the membrane-bound state, Trp fluorescence was measured in water (Figure 4A) and in small unilamellar vesicles (SUVs) before and after the treatment with chymotrypsin (Figure 4B), a proteolytic enzyme that cleaves peptide bonds involving hydrophobic amino acids. In water, we observed that the Trp residues are completely exposed to the aqueous solution, and after the addition of the proteolytic enzyme, we observed an increase in Trp fluorescence (Figure 4A), likely due to the breaking of interactions among molecules in water, which allows enhanced accessibility to acrylamide. Instead, a different behavior was recorded in SUVs (Figure 4B). In both conditions, we observed that Temporizin-1 is inserted into the membrane, and after the addition of chymotrypsin, the Trp fluorescence is changed significantly in the eukaryotic SUVs in comparison with that recorded in protozoan SUVs. While the fluorescence slightly increased in protozoan SUVs, suggesting that the Trp residues are buried and protected against enzymatic digestion, in eukaryotic SUVs, the fluorescence enhancement indicated that Trp residues are probably located at the bilayer–water interfacial region, making them accessible to the enzyme.

### 2.4. Binding Analysis by Bio-Layer Interferometry (BLI)

**Figure 5:**
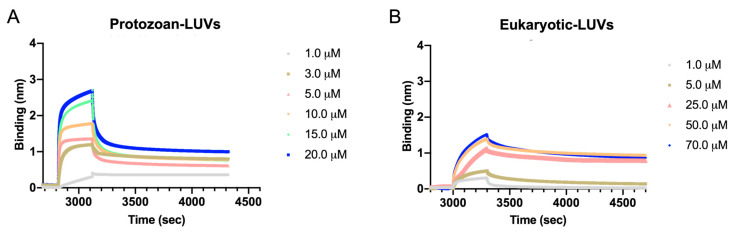
Association and dissociation kinetics of Temporizin-1 with LUVs immobilized on an APS sensor. Binding was assessed using Temporizin-1 at concentrations ranging from 1 to 20 μM for protozoan LUVs (**A**) and from 1 to 70 μM for eukaryotic LUVs (**B**).

To better understand the affinity of Temporizin-1 with protozoan and eukaryotic membranes, we performed the analysis by Bio-Layer Interferometry (BLI) and using APS biosensors to immobilize protozoan and eukaryotic LUVs. For this approach, it was necessary to obtain liposomes with the same mean size and low polydispersity index, indicating a monodisperse solution, so that the liposome immobilization can be correlated exclusively to liposome composition. The sensorgrams shown in Figure 5 display the binding profiles of Temporizin-1 showing a clear, concentration-dependent binding to protozoan and eukaryotic LUVs. The sensorgrams reported in Figure 5A exhibited a rapid association phase followed by a relatively slow dissociation, suggesting a strong and potentially stable interaction and high affinity with the protozoan-like membrane. Additionally, concentrations above 20 μM were not tested, as peptide aggregation on the liposome surface led to signal disturbance.

Moreover, although a concentration-dependent association is still observed, the binding response of Temporizin-1 to eukaryotic LUVs is significantly lower, suggesting a lower affinity of Temporizin-1 with this membrane type (Figure 5B). Specifically, higher peptide concentrations were required for eukaryotic LUVs; in fact, the peptide produced a weaker binding signal compared to protozoan LUVs under identical conditions. This indicates that the membrane interaction is stronger with protozoan LUVs. We used numerical integration analysis that exploits nonlinear analysis to fit an integrated rate equation directly to the sensorgrams, fitting the peptide sensorgrams with the simplest 1:1 Langmuir binding model, allowing the estimation of dissociation constant (KD) values equal to 1.7 ± 0.9 µM and 62.1 ± 8.2 µM for protozoan and eukaryotic LUVs, respectively. The fits yielded R^2^ values close to 1, although this model may not fully capture potential multivalent interactions or peptide aggregation. Overall, these results highlight Temporizin-1’s preferential binding to protozoan-like LUVs over eukaryotic ones.

### 2.5. Peptide-Induced Aggregation and Membrane Perturbations

**Figure 6:**
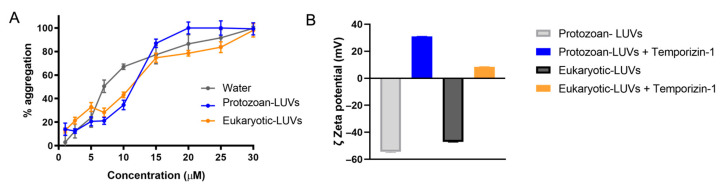
The aggregation percentage of Temporizin-1 measured in water and in LUVs (panel (**A**)) by ThT assay, and the zeta potential recorded in both liposomal conditions by DLS analysis (panel (**B**)). Data represent the mean of three independent experiments.

To further clarify the mechanism of interaction of Temporizin-1 with protozoan and eukaryotic LUVs used as model systems, we tested the ability of Temporizin-1 to aggregate in water and into protozoan and eukaryotic LUVs using Thioflavin T (ThT) [34]. As observed in Figure 6A, Temporizin-1 has a strong propensity to aggregate in all conditions. The peptide displayed a tendency to aggregate in water, where it is already aggregated for ~65% at the concentration of 10 μM. Upon interaction with LUVs, Temporizin-1 reached complete oligomerization at different concentrations depending on the membrane type. In particular, the full aggregation was achieved at 20 μM in protozoan LUVs, whereas in eukaryotic LUVs, complete oligomerization was obtained only at the highest tested concentration of 30 μM (Figure 6A).

To further investigate the interaction of the peptide with the membrane surface, we measured the zeta potential of our liposome models in the absence and presence of Temporizin-1 by performing Dynamic Light Scattering (DLS). As reported in Figure 6B, the zeta potential of protozoan LUVs shifts dramatically after incubation with 50 μM Temporizin-1, changing from −54 mV to +31 mV. Similarly, the zeta potential of eukaryotic LUVs shifts from −47 mV to +8 mV upon peptide treatment.

**Figure 7:**
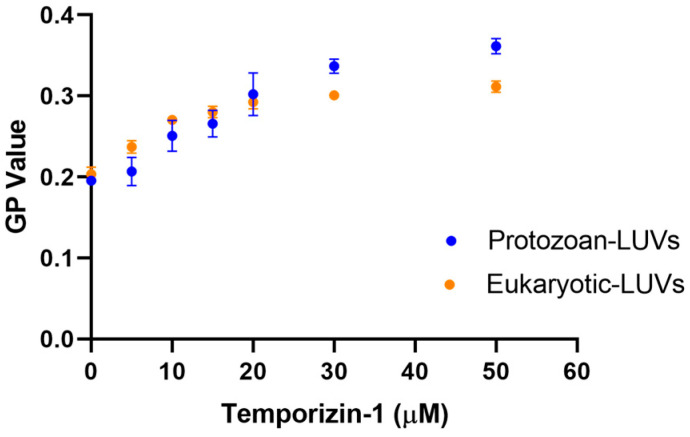
Membrane fluidity evaluation using the GP value in function of Temporizin-1 concentration.

To evaluate the effect of Temporizin-1 on membrane fluidity, we performed a Laurdan-based fluorescence assay [35]. Laurdan is a polarity-sensitive dye that incorporates into lipid bilayers and reports on membrane packing by changes in its emission spectrum. We measured the Generalized Polarization (GP) values of liposomes before and after peptide treatment. A decrease in GP values indicates a more disordered membrane, while an increase suggests a more ordered membrane. As shown in Figure 7, Temporizin-1 slightly increases the GP values in both protozoan and eukaryotic LUVs, with a more pronounced effect in protozoan membranes at the highest concentration of 30 μM and 50 μM, suggesting a partial modification of the membrane order and a resulting decrease in membrane fluidity.

### 2.6. Insights into the Secondary Structure of Temporizin-1

To understand the secondary structure of Temporizin-1, Circular Dichroism (CD) spectra were collected in water, in 2,2,2-Trifluoroethanol (TFE), and in protozoan and eukaryotic LUVs.

**Figure 8:**
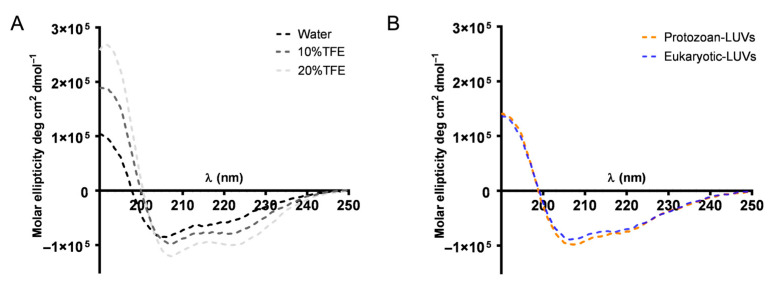
CD spectra of Temporizin-1 in water and in TFE (**A**), and in two different liposomes (**B**).

The CD spectra reported in Figure 8A show that Temporizin-1 in water already exhibits minima at ~208 and ~222 nm, which are typical of an α-helical conformation. In the presence of TFE (10% and 20%), the two minima become more pronounced. Similarly, in both protozoan and eukaryotic SUVs (Figure 8B), the peptide adopts an α-helical conformation, although the ratio between the two minima suggests the presence of aggregates.

In addition, the secondary structure of Temporizin-1 was studied by Nuclear Magnetic Resonance (NMR) in pure water, and in micelle environment by Sodium Dodecyl Sulfate (SDS) and Dodecylphosphorylcholine (DPC). SDS micelles carrying a strong negative charge mimic the protozoan membrane that presents a high content of anionic phospholipids, whereas the DPC micelles are zwitterionic and mimic the eukaryotic membrane rich in zwitterionic lipids [36,37].

**Figure 9:**
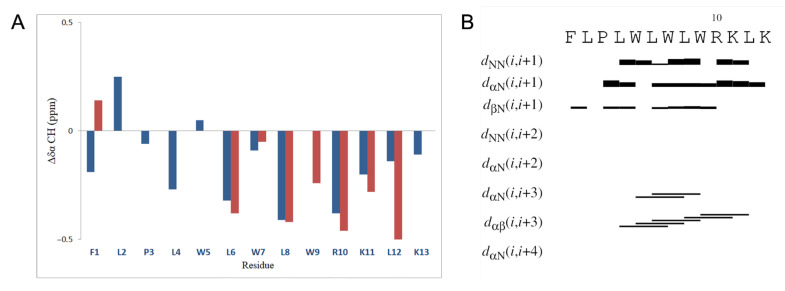
Chemical shift deviations from random coil values of αCH protons of Temporizin-1 in H2O/D2O 90/10 *v*/*v* (blue bars) and in DPC 50 mM (red bars) (**A**). Most relevant NOE contacts of Temporizin-1 in H2O/D2O 90/10, *v*/*v* (**B**).

As observed by NMR studies, Temporizin-1 is perfectly soluble and organized in a helix structure, as also evidenced by CD studies. Indeed, both the chemical shift values of the backbone αCH protons (Figure 9A) [38,39] as the measured pattern of NOEs (Figure 9B) are in accordance with predicting a helix structure localized in the L4-L12 segment of the peptide.

**Figure 10:**
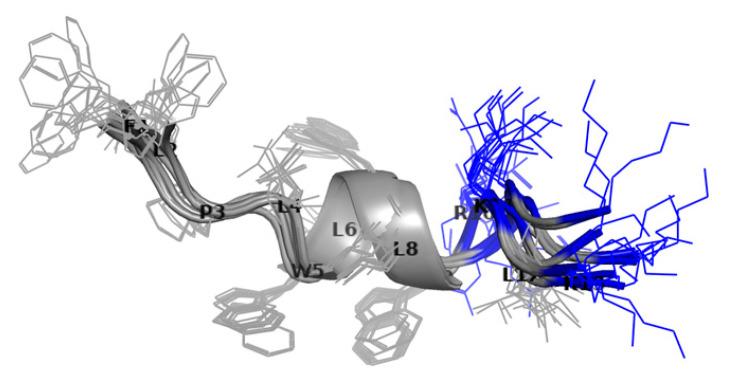
Cartoon representation of the best 15 NMR/CYANA structures, clustered by Chimera, of Temporizin-1 in H_2_O/D_2_O. Side-chains are shown as sticks colored by amino acid type: gray, hydrophobic; blue, basic. bb RMSD: 0.99 ± 0.26 Å; heavy RMSD: 1.75 ± 0.33 Å. PDB code 9S5G.

The three-dimensional structure determination of Temporizin-1 was performed with the CYANA [40], using as the upper limit (upl) of inter-proton distances 194 NOE-derived distances (161 intra-residues, 26 sequential, 7 long-range). Detail statistics were summarized in Table S1. The first ranked CYANA structures, i.e., those with the lowest target function values, were grouped by resemblance by Chimera-UCSF [41] and used to represent the peptide conformation (Figure 10). The atomic coordinates are deposited in the Protein Data Bank (PDB code 9S5G).

The backbone superimposition of the best 15 structures shows a helix arrangement in the L4-L2 segment that becomes strictly canonical in the W5-L8 central segment (here, the RMSD value on the backbone atoms is 0.26 Å). It is worth noticing that in this arrangement, W5 and W9 aromatic bicyclic rings are well oriented to give stacking ability and thus stabilize the helix structure.

**Figure 11:**
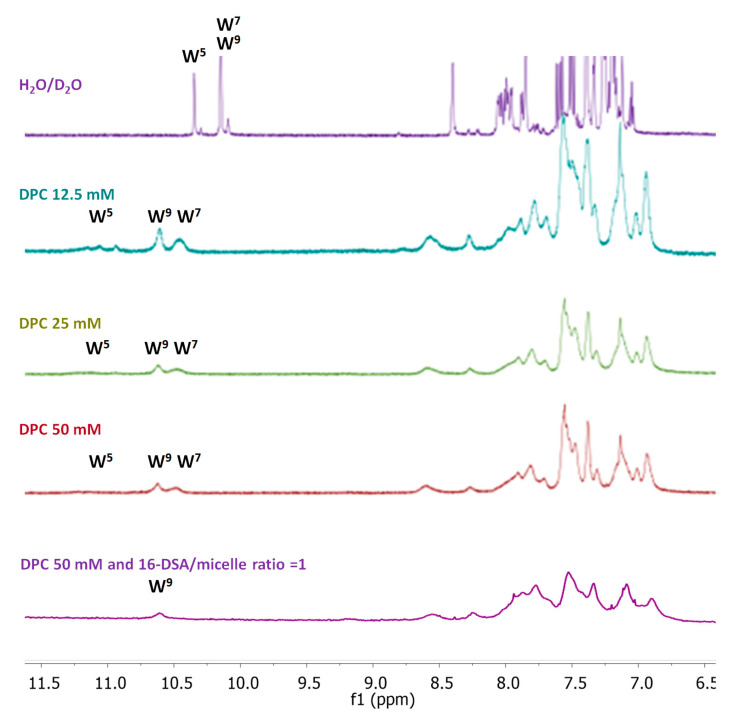
Comparison of low field region of proton NMR spectra acquired for Temporizin-1 in H_2_O/D_2_O (purple) and at increasing amounts of DPC monomer: 12.5 mM (blue), 25 mM (green), 50 mM (red). The bottom spectrum (magenta) is acquired in DPC 50 mM at 16-DSA/micelle ratio = 1. The spectra show changes in both the chemical shift and linewidth of the indole NH proton of W^5^, W^7^, and W^9^ residues.

Furthermore, NMR spectroscopy has been useful in clarifying structural details of the interaction between Temporizin-1 and mimic membranes such as SDS and DPC micelles. However, we found that the interaction of Temporizin-1 with SDS micelles cannot be studied by NMR due to insolubility phenomena under the experimental conditions explored (Temporizin-1 0.8 mM, SDS monomer in the range of 25–150 mM). On the other hand, the Temporizin-1/DPC micelle system has been well characterized via NMR. Temporizin-1 binds the DPC micelles and preserves the helix structure. The 1D 1H spectra of the Temporizin-1 in plain water as well as in DPC micelles are shown in Figure 11.

The addition of DPC micelles causes a broadening of signals, indicating that the peptide interacts with micelles. The proton spectrum showed no further change after reaching the DPC monomer/peptide molar ratio R of 94 corresponding to 50 mM DPC, DPC micelle/peptide ratio ca. 3 (Figure 11). In these conditions, the deep interaction between the peptide and the micelles causes the disappearance of some resonances and a reduction in the intensities of several others. It is interesting to note that the line widths of the indole protons of W5, W7, and W9 act as probes to detect the peptide orientation with respect to the micelle. In fact, in the presence of DPC, the signal of W5 is so broad that it disappears, while that of W7 shows a broader linewidth compared to that of the W9 signal. These results alone suggest that the peptide dives into the micelle through the N-terminal end. The structure adopted by the peptide in the bound state was made only by evaluating αCH deviations from random coil values (Figure 9A, red bars), and these deviations confirm the helical conformation already observed in pure water [38,39].

In addition, to investigate the insertion of Temporizin-1 whitin DPC micelles, the spin label 16-doxyl stearic acid (16-DSA) was added to the peptide/DPC samples. Since 16-DSA readily partitions into DPC micelles [42], it serves as a probe for identifying membrane-embedded residues [42]. The unpaired electron of 16-DSA induces paramagnetic relaxation, resulting in line broadening of signals of protons spatially close to the spin label. It can be observed in Figure 11 (bottom spectrum) that the presence of 16-DSA clears the signal of the indole NH proton of W7 while broadening that of W9. Indeed, the resonances of the N-terminal residues, already poorly observable in the absence of the paramagnetic substance, disappear completely, while the resonances of C-terminal residues, those not immersed in the membrane, show residual intensities. A plot of the residual percentage of the intensities (I) of NH-αCH cross peaks (I_16-DSA_/I_0_) measured for the last C-terminal residues of Temporizin-1 in two TOCSY spectra acquired with (I_16-DSA_) and without (I_0_) 16-DSA are reported in Figure S1. Overall, our results indicate that Temporizin-1 dives into the DPC micelle with its N-terminal hydrophobic portion, while the positively charged C-terminal portion remains anchored on the surface.

### 2.7. Biological Activity of Temporizin-1

The biological behavior of Temporizin-1 was evaluated performing in vitro studies on both eukaryotic and protozoan cells. Firstly, the cytotoxicity effects of Temporizin-1 were evaluated in Vero cells by measuring intracellular ATP levels using the CellTiter Glo reagent (Promega, Milan, Italy) after 72 h of exposure to various concentrations (3.12–100 µM). The calculated CC_50_ value was 27.1 ± 1.9 µM at 72 h, consistent with the strong interaction of Temporizin-1 with eukaryotic membranes as observed in biophysical experiments. In comparison, benznidazole (Bz) exhibited minimal cytotoxicity, with CC_50_ values exceeding the maximum tested concentration (500 µM), as detailed in Table 1.

The antiparasitic activity of Temporizin-1 was assessed against the trypomastigote and intracellular amastigote forms of the Dm28c-Luc clone of *T. cruzi*, which expresses luciferase, allowing for the accurate measurement of parasite viability through luminescence detection. In trypomastigote assays, Temporizin-1 demonstrated superior activity compared to Bz, with an IC_50_ value of 7.2 ± 0.2 µM, effectively making it about twice as potent as Bz (IC_50_ = 18.6 ± 1.5 µM), as described in Table 1. However, the cytotoxic effects of Temporizin-1 impacted its selectivity index (SI), which was estimated at 3.8. In contrast, Bz demonstrated a significantly higher selectivity due to its lower toxicity profile in Vero cells, yielding an SI greater than 26.8 (Table 1), which indicates a wider therapeutic margin for the reference drug.

The effect of Temporizin-1 on intracellular amastigotes was assessed using Vero cells monolayers infected with *T. cruzi* Dm28c-Luc. Following a 72 h treatment with increasing concentrations of Temporizin-1, parasite viability was measured by luminescence. The findings revealed that Temporizin-1 exhibited an IC_50_ of 1.9 ± 0.4 µM, demonstrating comparable potency to Bz, which had an IC_50_ of 2.4 ± 1.2 µM against intracellular amastigotes. However, the selectivity index (SI) indicated a much broader therapeutic margin for Bz (SI > 208.3) relative to Temporizin-1 (SI = 15.5), as shown in Table 1.

**Figure 12:**
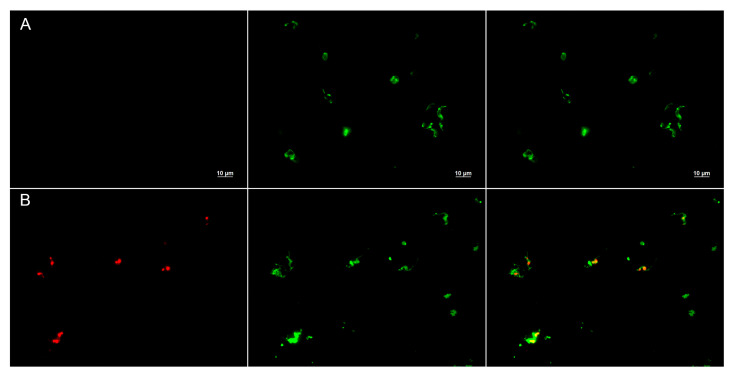
Comparison of membrane permeabilization of trypomastigotes untreated (panel (**A**)) and treated (panel (**B**)) with Temporizin-1 (10 µM) for 24 h. Parasites were stained with the DNA-binding fluorescent probe propidium iodide to detect membrane damage and Concanavalin A-FITC to visualize morphological alterations of the parasite membrane.

Additionally, the effect of Temporizin-1 on the plasma membrane integrity of *T. cruzi* was assessed using propidium iodide (PI) staining. Parasites treated with the peptide at 10 μM exhibited a marked increase in PI fluorescence compared to the untreated control group. Fluorescence microscopy revealed intense staining localized in both the nucleus and the kinetoplast, indicating PI internalization due to compromised membrane integrity (Figure 12). In contrast, untreated parasites showed minimal fluorescence, consistent with intact plasma membranes that exclude PI. These findings demonstrate that the peptide induces membrane permeabilization, leading to loss of selective barrier function and allowing PI entry into intracellular compartments.

### 2.8. Assessment of Synergistic Interaction Between Temporizin-1 and Bz

**Figure 13:**
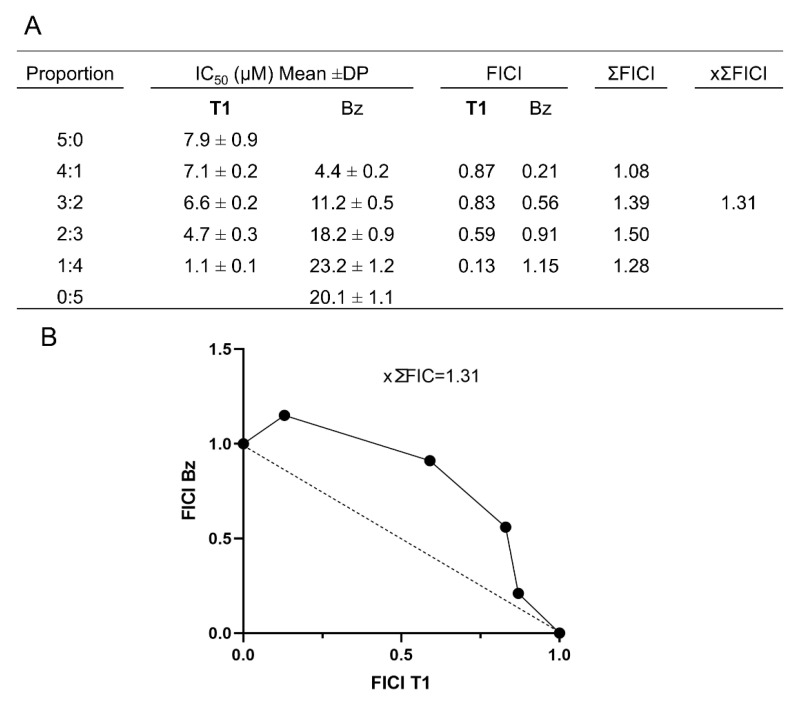
Analysis of the in vitro combinatorial activity of Temporizin-1 (T1) with benznidazole (Bz) against trypomastigote forms of *T. cruzi* (**A**). The interactions of these treatments were quantified using the fractional inhibitory concentration index (FICI), derived from the IC50 values of each agent. Isobologram plots were constructed from the FICI values, with the dashed line representing the threshold for additive interaction between the compounds under evaluation (**B**).

We also evaluated the efficacy of a combined treatment with Temporizin-1 and Bz against trypomastigote forms of *T. cruzi* (Dm28c-Luc). The membranolytic activity of Temporizin-1 can promote the uptake of Bz and increase its efficacy at low concentrations. In this assay, various combinations were prepared with Temporizin-1–Bz ratios (5:0, 4:1, 3:2, 2:3, 1:4, and 0:5). The IC_50_ values for each combination were used to calculate the fractional inhibitory concentration index (FICI), as previously described (Figure 13A). The data show that the average of the sum of the FICIs (×∑FICI = 1.31) indicates an additive interaction between the compounds tested, suggesting that the combined effect reflects the sum of the individual effects. The FICI values and the corresponding isobologram plot are shown in Figure 13B.

## 3. Discussion

AMPs represent a promising strategy to counteract antibiotic resistance, as their primary mechanism of action involves direct interaction with and subsequent disruption of microbial membranes. The physicochemical properties including hydrophobicity, amphipathicity, and net positive charge, control the AMP’s behavior towards the membrane [43]. The net positive charge is implicated in the membrane anchoring by establishing electrostatic interactions, while the hydrophobicity plays a key role in the insertion and incorporation of AMPs inside the lipid core [44].

Herein, we explored the potential membranolytic activity of Temporizin-1, a synthetic hybrid peptide derived from the Temporin A, Gramidicin, and poly-leu peptides, which displayed strong activity on *T. cruzi* and moderate cytotoxicity against mammalian cells [25].

Firstly, we determined the physicochemical properties of Temporizin-1, including amphipathicity and hydrophobicity, which is featured by a net positive charge of +4 essential for electrostatic interactions with negatively charged phospholipids, and a repeated motif WL conferring hydrophobicity and the ability to incorporate inside the lipid core. The intrinsic peptide hydrophobicity is evidenced by its hydrophobic moment (0.242), aliphatic index (150.0), and GRAVY score (0.400), parameters that highlight its amphipathic nature and potential membrane activity. All these theorical parameters were obtained by HeliQuest and ExPASy, while the Wimley–White hydrophobicity scale was calculated from the MPEx software obtaining the negative Gibbs energy of −9.09 Kcal·mol^−1^ and suggesting a membrane insertion of Temporizin-1 [45].

Moreover, these physicochemical properties endow Temporizin-1 with the capacity to interact with and insert into lipid membranes, leading to membrane disruption. To investigate its mechanism of action, herein, we employed liposomes designed to mimic both the lipid composition of protozoan and eukaryotic membranes and conducted a series of biophysical assays. These two-membrane model systems enable a comparative analysis of membrane interactions, providing insights into potential selective targeting based on lipid composition differences.

By performing the lipid mixing and leakage assays, we observed strong membranolytic activity of Temporizin-1 both in the presence of protozoan and eukaryotic LUVs. In both conditions, the peptide induced a complete membrane fusion, but at different peptide/lipid ratios. Specifically, Temporizin-1 induced the complete fusion of eukaryotic LUVs already at the low P/L ratio of 0.05; in contrast, in protozoan-mimetic LUVs, Temporizin-1 showed a gradual increase in lipid mixing, reaching the full membrane fusion at a higher P/L of 0.15.

Additionally, Temporizin-1 exhibited pronounced membranolytic activity as evidenced by its ability to induce leakage both of protozoan and eukaryotic LUVs. When we performed the LUVs’ titration with Temporizin-1, we observed a complete protozoan LUVs’ disruption at a P/L ratio of 0.2, measuring a significant calcein release from the LUVs. These findings are consistent with previous studies reporting that Temporizin-1 can induce pore formation in the *Trypanosoma* membrane, likely due to the presence of the gramicidin-derived sequence. Furthermore, the significant leakage observed in eukaryotic LUVs at all P/L ratios highlights the cytotoxic potential of Temporizin-1 previously reported [25], where the pore formation was observed in mammalian cells but at lower concentrations compared to the native peptide Temporizin.

Once the membrane action of Temporizin-1 was ascertained, we accomplished a systematic study to understand how the physicochemical properties can influence the peptide affinity and interaction with membranes featured by different compositions. Firstly, the presence of repeated WL motifs can favor the insertion of Temporizin-1 into the hydrophobic core of lipid bilayers. Tryptophan residues are particularly important in this process, as they show a strong preference for the interfacial regions of lipid membranes and thus facilitate AMP insertion [46]. In our study, we confirmed Trp insertion by performing fluorescence quenching experiments with acrylamide, a quencher unable to interact with lipids and which prefers the aqueous solution. While the acrylamide is able to strongly quench Temporizin-1 in water, in protozoan and eukaryotic LUVs, the Trp residues are less accessible because they are buried inside the lipid core. Interestingly, these results were further supported by calculating the Stern–Volmer constants (K_sv_). The high Ksv value (8.9 ± 0.3) of Temporizin-1 in water confirmed the Trp accessibility to acrylamide in this condition, whereas the low K_sv_ values, 0.81 ± 0.05 and 1.9 ± 0.1 for protozoan and eukaryotic LUVs, demonstrated the reduced Trp accessibility. This slight difference between K_sv_ values obtained in LUVs highlighted a potential different penetration of Temporizin-1 in the membrane. To investigate this aspect, we performed proteolytic cut with chymotrypsin following peptide incubation with SUVs. If the Trp residues are at the interface, the enzyme recognizes and cuts them, resulting in the enhancement of Trp fluorescence; conversely, when Trp residues are located inside the core, the enzyme is not able to cut, and an increase in fluorescence is not recorded. Interestingly, we observed that Temporizin-1 is completely incorporated inside the protozoan SUVs, while in eukaryotic SUVs, the peptide is probably partially inserted inside the lipid bilayer and located at the membrane interface since the Trp fluorescence emission increased significantly after the proteolytic cut. This different behavior could be attributed to the different lipid composition of membranes. Indeed, eukaryotic membranes, rich in zwitterionic lipids, could favor a more interfacial peptide localization, whereas protozoan membranes, enriched in anionic lipids, promote stronger electrostatic interactions with Temporizin-1, driving Trp residues deeper into the bilayer and reducing their enzymatic accessibility.

Moreover, this different membrane interaction was also further investigated by BLI experiment using APS biosensors to immobilize protozoan and eukaryotic LUVs. Temporizin-1 showed a strong interaction and high affinity with the protozoan-like membrane, while its interaction with eukaryotic LUVs was comparatively weaker. This result was also supported by KD values calculated using the Langmuir binding model, presenting values of 1.7 ± 0.9 µM for protozoan LUVs and 62.1 ± 8.2 µM for eukaryotic LUVs, thereby confirming the weaker affinity of Temporizin-1 for eukaryotic LUVs.

Additionally, the effect on the membrane due to peptide interaction and insertion was investigated in terms of zeta potential, aggregation, and membrane fluidity.

Through the DLS analysis, we observed a strong binding between positively charged Temporizin-1 and protozoan LUVs, where the zeta potential changed from −54 mV to +31 mV. Similarly, this variation was also measured in the presence of eukaryotic LUVs. These significant changes indicate a strong electrostatic interaction between Temporizin-1 and the surface of both types of LUVs driven by the peptide’s net positive charge of +4. Additionally, upon the LUVs’ interaction, the peptide showed a strong propensity to aggregate and change the membrane fluidity both in the presence of protozoan and eukaryotic LUVs. ThT fluorescence measurements revealed a concentration-dependent aggregation, with progressive oligomerization occurring between 2.5 and 50 µM and complete oligomer formation observed at the highest concentrations. Notably, the peptide already displayed partial aggregation (~65%) in aqueous solution at 10 µM, indicating an intrinsic propensity to oligomerize. In the presence of protozoan LUVs, complete aggregation was achieved at 20 µM, whereas in eukaryotic LUVs, full oligomerization was only observed at 30 µM.

In these same conditions, we also observed an increase in the GP value by recording the Laurdan probe emission in both liposomal compositions, indicating a partial modification of the membrane order and a resulting decrease in membrane fluidity, likely due to extensive aggregation on the membrane surface.

In addition, we also studied the conformational change occurring when AMPs meet the membrane. Generally, AMPs are in a disordered or partially structured state in water, and upon the membrane binding, electrostatic attraction to negatively charged lipid headgroups and hydrophobic interactions with the lipid core can induce folding into structured conformations, typically α-helices or β-sheets [47]. Interestingly, Temporizin-1 adopts a structured conformation even in aqueous solution. The presence of an α-helical structure plays a crucial role in promoting peptide interactions with target membranes and enabling membrane disruption [48]. In this amphiphilic arrangement, the cationic and hydrophobic residues are segregated on opposite faces of the helix, thereby facilitating the interaction of antimicrobial peptides (AMPs) with membranes [49]. The CD spectrum evidenced the presence of a stable helical structure that is kept after the membrane interaction, as shown by CD spectra recorded in the presence of LUVs. The CD data were further confirmed by NMR analysis. The NMR provided the experimental structure of free Temporizin-1 in water, confirming the molecule’s ability to interact with mimetic micelles, and determined the way the peptide interacts with the micelle. The free state sequence adopts a helical conformation in the L4-L12 segment. Temporizin-1’s ability to interact with micelle systems was analyzed using DPC, a monomer whose aggregation produces micelles of moderate size and is therefore compatible with the observation technique. The Temporizin-1/DPC micelles interaction is confirmed by both the change in chemical shifts and the broadening of the peptide’s spectral signals. This last observation is due to the fact that in its micelle-bound state, the peptide adopts longer rotational correlation times than in its free state, resulting in increased nuclear relaxation rates and signal broadening. This phenomenon also allowed us to deduce the geometry of the peptide–micelle interaction. We can observe how the indole NH proton signals of W5, W7, and W9 shift from their position in the free peptide and that they all broaden, albeit to varying degrees. While the W5 signal broadens so much that it almost disappears, the corresponding signal of W7 broadens less, and that of W9 even less. This ordered pattern of signal widening that sequentially decreases in the C-terminal direction indicates that Temporizin-1 dives into the core of the micelle with its hydrophobic N-terminal portion. This observation is consistent with the effects produced by the addition of 16-DSA to the Temporizin-1 micelle solution, a radical species that penetrates the micelle and “turns off” the resonances of the most internalized portions of the peptide.

Overall, our biophysical results were supported by biological studies demonstrating that Temporizin-1 exerts significant activity against the infective forms of *T. cruzi*, in addition to previously reported effects on non-infective epimastigotes [25]. Notably, Temporizin-1 displayed high selectivity against intracellular amastigotes, with a selectivity index of 15.5. This result is consistent with the strong interaction with the eukaryotic membrane, where its effect consists of the membrane disruption with the ability to reach and kill intracellular amastigotes.

However, biophysical and biological studies on the mode of action demonstrate that Temporizin-1 induces membrane permeabilization, allowing PI entry into intracellular compartments; however, the selective action of the peptide against intracellular forms suggests additional mechanisms, including induction of apoptosis, mitochondrial damage, DNA fragmentation, and immunomodulatory activity [50].

Moreover, the combination of Temporizin-1 with the traditional drug Bz has determined an additive effect (as shown by ×∑FICI ≤ 0.5) that could enhance treatment efficacy by reducing drug dosages, shortening overall treatment duration, and mitigating adverse effects [51]. This observation aligns with our biophysical findings, suggesting that Temporizin-1 may facilitate drug uptake through perturbation of the protozoan membrane, thereby contributing to the observed synergistic effect.

Based on our biophysical and biological studies, we demonstrated membranolytic activity of Temporizin-1, supporting a potential toroidal or barrel stave mechanism both on protozoan and eukaryotic LUVs. Nevertheless, additional intracellular mechanisms contributing to the peptide’s potential activity within the cell cannot be ruled out. Moreover, the combination of Temporizin-1 with conventional drugs may represent a promising strategy to restore the efficacy of agents that have lost clinical utility due to the development of pathogen resistance.

## 4. Materials and Methods

### 4.1. Materials

All conventional N^α^-Fmoc-amino acids were acquired from GL Biochem Ltd. (Shanghai, China). Fmoc-Rink amide resin, *N,N*-diisopropylethylamine (DIEA), piperidine, ergosterol (ERG), and trifluoroacetic acid (TFA) were purchased from Iris-Biotech GMBH (Marktredwitz, Germany). Oxyma pure, *N,N*′-Diisopropylcarbodiimide (DIC), 1-[Bis(dimethylamino)methylene]-1H-1,2,3-triazolo [4,5-b]pyridinium 3-oxid hexafluorophosphate (HATU), triisopropylsilane (TIS), calcein, Thioflavin T, Laurdan, acrylamide, α-chymotrypsin from bovine pancreas, Deuterium oxide (D_2_O) (99.9% isotopic purity), dimethyl sulfoxide (DMSO-d_6_) (99.9% isotopic purity), Dodecylphosphorylcholine-d_38_ (DPC-d_38_) (98% isotopic purity), and 16-doxylstearic acid were purchased from Merck (Milan, Italy). Phospholipids: phosphatidylcholine (PC), phosphatidylethanolamine (PE), phosphatidylinositol (PI), sphingomyelin (SM), phosphatidylserine (PS), Rhodamine and 12-(N-methyl-N-(7-nitrobenz-2-oxa-1,3-diazol-4-yl)) (NBD)–phosphatidylethanolamine (Rho-PE and NBD-PE, respectively), and cholesterol (Chol) were purchased from Avanti Polar Lipids (Alabaster, AL, USA). Sodium-3-(trimethylsilyl) propionate 2,2,3,3-d4 (TSP) was from Cambridge Isotope Laboratories (CIL), Inc. (Andover, MA, USA).

### 4.2. Peptide Synthesis

Temporizin-1 was synthesized using the Fmoc/*t*Bu approach via solid-phase peptide synthesis (SPPS) [52]. The synthesis was performed using an Fmoc-Rink amide resin (0.67 mmol/g). During the synthesis, the Fmoc removal was performed using two repeated 10 min treatments with 20% piperidine in DMF under shaking at room temperature (rt). The coupling reaction was carried out in two different steps: (i) Fmoc-AA (2 equiv), DIC (2 equiv), and Oxyma Pure (2 quiv), in DMF as solvent for 25 min at rt; (ii) Fmoc-AA (2 equiv), HATU (2 equiv), DIPEA (4 equiv), in DMF for 25 min at rt. Once the final peptide-chain was assembled, Temporizin-1 was cleaved from the resin along with the side-chain protecting groups by treating the resin with a cleavage mixture of TFA:TIS:H_2_O (95:2.5:2.5, *v*/*v*/*v*) for 3 h at rt. Then, the peptide was purified by preparative reverse-phase high-performance liquid chromatography (RP-HPLC) using a Phenomenex Kinetex C18 column and a linear gradient of MeCN (0.1% TFA) in water (0.1% TFA) from 10% to 70% over 35 min. Peptide identity was determined by electrospray ionization mass spectrometry (ESI-MS), and purity was assessed by analytical HPLC (Jasco LC-4000, Cremella, Italy) (Figures S2 and S3).

### 4.3. Liposome Preparation

Large unilamellar vesicles (LUVs) and small unilamellar vesicles (SUVs), consisting of PC:PE:PI:SM:PS:ERG (35:30:15:5:10:5, ratio in moles) and PC:Chol (70:30, ratio in moles), mimic the protozoan and eukaryotic membranes, respectively. LUVs were prepared using the extrusion method, while SUVs were obtained through ultrasonication. Firstly, we prepared the lipid film by dissolving each phospholipid in chloroform and mixing them with each other at a specific ratio as reported above. Then, the chloroform was removed under nitrogen, and the lipid film was lyophilized overnight. For the LUVs’ preparation, the lipid film was hydrated with water or buffer for 1h and was then freeze–thawed 6 times and extruded 10 times through polycarbonate membranes with 0.1 mm diameter pores [53]. In all experiments, LUVs were characterized for their average size and polydispersity index (PDI) measured by the Dynamic Light Scattering (DLS) analysis. Specifically, the average size of protozoan and eukaryotic LUVs was 117.4 ± 0.8 nm (PDI = 0.14 ± 0.01) and 124.9 ± 0.3 nm (PDI = 0.15 ± 0.02), respectively. Instead, for the SUVs preparation, the suspension was sonicated for 30 min after the lipid hydration. The average size of protozoan and eukaryotic SUVs was 80.5 ± 0.8 nm (PDI = 0.21 ± 0.01) and 82.5 ± 0.5 nm (PDI = 0.17 ± 0.02), respectively.

### 4.4. Lipid Mixing Assay

The fusogenic activity of Temporizin-1 was evaluated via fluorescence resonance energy transfer (FRET), using two distinct sets of liposomes, including unlabeled and labeled LUVs [54]. The liposomes were labeled using NBD as donor and Rho as acceptor probe. We prepared unlabeled LUVs mimicking protozoan membranes by mixing phospholipids PC:PE:PI:SM:PS:ERG (35:30:15:5:10:5, ratio in moles), at a final lipid concentration of 0.16 mM, while labeled LUVs PC:PE:PI:SM:PS:ERG were prepared with 0.6 mol% NBD-PE and 0.6 mol% Rho-PE, at a final lipid concentration of 0.04 mM [55]. To evaluate membrane mixing, unlabeled and labeled LUVs were mixed at a ratio of 4:1 to obtain a final lipid concentration of 0.1 mM, while Temporizin-1 was added to LUVs at increasing concentrations at lipid/peptide ratios of 0.01, 0.03, 0.05, 0.1, 0.15, and 0.20. The lipid mixing was assessed measuring the NBD emission at 530 nm and Rho emission at 590 nm with an NBD excitation at 465 nm [56]. To quantify membrane fusion, the percentage of fusion was calculated after each peptide addition. This was normalized against a reference value of 100%, which corresponded to maximal lipid mixing upon the addition of Triton X-100 (0.05% *v*/*v*). The equation for calculating the percentage of fusion is as follows:

$$\frac{\left[F530/F590\right]peptide-[F530/F590]blank}{\left[F530/F590\right]triton-[F530/F590]blank}\;\times \;100$$

where F530 and F590 represent the intensity at 530 nm and 590 nm, respectively, which we calculated in the presence and absence of the peptide and triton.

### 4.5. Trp Quenching by Acrylamide

The incorporation of tryptophan residues inside the lipid bilayer was investigated by performing their quenching with acrylamide. Acrylamide is a water-soluble quencher unable to enter lipid bilayers, and this makes it well-suited to detect Trp residues exposed to an aqueous environment [57]. The experiment was performed in buffer solution and in LUVs mimicking protozoan and eukaryotic membranes (lipid final concentration = 250 µM). Temporizin-1 was dissolved at 10 µM in water or incubated with LUVs and then the quenching was performed at increasing acrylamide concentrations from 0.02 M to 0.22 M [58]. The Trp fluorescence spectrum was obtained exciting at 295 nm. The data were analyzed using the Stern–Volmer equation: F_0_/F = 1 + K_sv_[Q], where F_0_ and F correspond to the fluorescence intensities before and after the addition of the quencher (Q), respectively, and K_sv_ is the Stern–Volmer quenching constant, which correlates to the accessibility of the Trp residue to acrylamide.

### 4.6. Chymotrypsin Proteolysis Monitored by Fluorescence

The insertion of the Trp residue was assessed in the presence of α-chymotrypsin, both in buffer and small unilamellar vesicles (SUVs) mimicking protozoan and eukaryotic membranes. Chymotrypsin has a strong affinity towards aromatic amino acids and cuts peptide bonds involving Trp residues, resulting in an increase in Trp fluorescence [59]. In the buffer condition, the peptide was dissolved at 10 μM in 100 mM Tris-HCl buffer (pH 7.3) and was then incubated with 3 μM chymotrypsin [60]. Fluorescence emission was monitored for 15 min using excitation and emission wavelengths of 295 and 355 nm, respectively. Spectra were collected before and after enzyme treatment. The same procedure was performed in the presence of SUVs (lipid final concentration = 250 uM). The peptide at 10 μM was incubated with SUVs for 10 min, and then the enzyme was added. The fluorescence emission of Trp was recorded under the same conditions over a 15 min period.

### 4.7. Leakage Assay

The ability of Temporizin-1 to induce the pore formation was evaluated by a calcein leakage assay. We prepared LUVs mimicking protozoan and eukaryote membranes loaded with calcein. Once the lipid film was prepared, it was hydrated with a solution of 10 mM Tris, 150 mM NaCl (pH 7.4), and 60 mM calcein for 1 h under stirring [33]. The liposome suspension was treated for obtaining LUVs, and then free calcein was removed by gel filtration using a Sephadex G-25 column (1.5 × 10 cm) at rt. Calcein release induced by the peptide treatment was monitored by measuring the fluorescence increase setting an excitation fluorescence at 485 nm and an emission at 535 nm. The data were normalized relative to the complete calcein release obtained by adding 0.1% Triton X to LUVs, which causes total liposome disruption. The percentage of leakage at different peptide concentrations was calculated using the formula % leakage = (F_i_ − F_0_)/(F_t_ − F_0_), where F_0_ represents the fluorescence of intact LUVs before the addition of the peptide, while F_i_ and F_t_ indicate the fluorescence intensity before and after the addition of the peptide and Triton X, respectively.

### 4.8. Bio-Layer Interferometry (BLI) Experiment

A Bio-Layer Interferometry (BLI) interaction assay was performed by employing the Sartorius Octet^®^ N1 System and APS Biosensors coated with aminopropylsilane (Sartorius Italy S.r.l., Grassina-Bagno a Ripoli (FI), Italy). Firstly, APS tips were hydrated in 1× PBS buffer for 600 s followed by equilibration for 300 s with PBS. Then, to immobilize the ligand, the tips were dipped, for 1800 s with 1000 rpm, into 200 µL liposome solution (5 mM) followed by wash step with PBS for 300 s to remove unbound vesicles. Afterwards, a blocking step with BSA (1 mg mL^−1^) was performed followed by another washing step with PBS for 300 s. Association of Temporizin-1 in a concentration range of 1–20 μM for protozoan LUVs and 1–70 μM for eukaryotic LUVs was performed for 300 s, while the dissociation was monitored for 1800 s with PBS [61]. All data analysis were processed using the Octet Analysis Studio 12.2 software (Sartorius, Göttingen, Germany). The data were fitted with a 1:1 Langmuir binding model, and the KDs were determined through the global analysis mode.

### 4.9. Peptide Oligomerization by Thioflavin T Assay

To detect peptide oligomerization in an aqueous solution and in LUVs mimicking protozoan and eukaryotic membranes, we used the fluorescent dye Thioflavin T (ThT) [62]. ThT strongly binds to aggregated structures, producing an increase in fluorescence emission at 482 nm and excited at 450 nm [63]. LUVs were prepared at 100 µM and hydrated with the buffer solution 100 mM NaCl, 10 mM Tris-HCl, 25 µM ThT, pH 7.4, for 1 h. The oligomerization was monitored by titrating LUVs with Temporizion-1 concentrations of 5, 2.5, 5, 7, 10, 15, 20, 25, 30, 40 and 50 µM. ThT fluorescence was recorded before and after the addition of the peptides using a Varian Cary Eclipse fluorescence spectrophotometer (Milan, Italy). The emission spectrum was measured at 482 nm upon excitation at 450 nm. Peptide aggregation was expressed as the percentage increase in ThT fluorescence according to the following equation:

$$\frac{\left(F_{f}-F_{0}\right)}{\left(F_{max}-F_{0}\right)}\times 100$$

where F_f_ indicates the fluorescence after peptide addition, F_0_ the initial fluorescence in the absence of peptides, and F_max_ is the fluorescence maximum obtained immediately after peptide addition.

### 4.10. Zeta Potential Measurement

Zeta potential measurements were carried out in the presence of protozoan and eukaryotic LUVs (final lipid concentration = 100 µM), and at peptide concentrations of 20, 30, and 50 µM. These measurements were conducted using a Zetasizer Nano-ZS (Malvern Instruments Ltd., Worcestershire, UK) equipped with a 4 mW He–Ne laser operating at 633 nm. Measurements were taken at a fixed backscattering angle of 173° and at a temperature of 25 °C. The results were performed in triplicate.

### 4.11. Laurdan-Based Assessment of Membrane Fluidity

To evaluate the effect of Temporizin-1 on membrane fluidity, we used the fluorescent probe Laurdan to observe phase transitions and measure variations in lipid packing, calculating the generalized polarization (GP) parameter [64]. Laurdan inserts into lipid bilayers and indicates the physical membrane state by altering its emission spectrum. The maximum emission wavelength at 490 nm indicated the liquid-disordered membrane phase, while the emission at 440 nm indicated the more liquid-ordered membrane phase. Lipid films mimicking both membranes were prepared at 100 uM and Laurdan was added to a final concentration of 5 µM. The lipid mixture was lyophilized and then hydrated with PBS 1× (pH 7.4) for 1 h under shaking. The LUVs were obtained by extrusion method as described above. Membrane fluidity was evaluated by incubating the LUVs with the peptide at concentrations from 5 µM to 50 µM. Laurdan emission spectra were recorded from 400 nm to 550 nm with excitation at 365 nm. Peptide-induced changes in membrane fluidity were quantified using the GP value, which is calculated as follows:

$$GP=\frac{I_{440}-I_{490}}{I_{440}+I_{490}}$$

where I_440_ and I_490_ indicate the fluorescence intensities at the maximum emission wavelength in the ordered and disordered phases, respectively.

### 4.12. Circular Dichroism Studies

The secondary structure of Temporizin-1 was studied by Circular Dichroism (CD) spectroscopy both in solution and in SUVs mimicking protozoan and eukaryotic membranes. CD spectra in water and 2,2,2-Trifluoroethanol (TFE) at 10% and 20% were recorded at 100 μM from 190 to 260 nm using a Jasco J-1500 CD Spectrometer (Cremella, Italy) with a quartz cell (0.1 cm) [65]. In membrane environments, SUVs (100 μM) were prepared and Temporizin-1 was added at the peptide/lipid ratio of 1. Each CD spectrum was recorded from 190 to 260 nm using a 0.1 cm quartz cell and converting the signal to molar ellipticity.

### 4.13. Nuclear Magnetic Resonance (NMR) Spectroscopy

Temporizin-1 was dissolved in 0.600 mL H_2_O/D_2_O (90/10, *v*/*v*) to achieve a peptide concentration of 0.5–0.6 mM for the NMR analysis. Incremental volumes (μL) of a DPC-d_38_ stock solution (1.0 M in H_2_O/D_2_O, 90/10 *v*/*v*, pH 6) were then added until no further changes were observed in the one-dimensional spectra. The NMR measurements in micellar environments were performed at DPC concentrations of 50 mM and peptide concentration of 0.55 mM for a DPC/peptide molar ratio R of 94. Assuming a micelle aggregation number of ∼30, the micelle/peptide molar ratio R is ∼ 3. NMR spectra were recorded on a Bruker 600 MHz Spectrometer (Department of Pharmacy—University “Federico II” of Naples) and equipped with a z-gradient 5 mm triple-resonance cryoprobe. One-dimensional proton (1D) and two-dimensional homonuclear (2D) NMR spectra were acquired at 298 K. Proton chemical shifts were referred to TSP (0.00 ppm). Two-dimensional TOCSY and NOESY were acquired with typical mixing times of 70 and 300 ms, respectively. Water resonance suppression was achieved using gradient methods. NMR spectra were analyzed by MESTRENOVA 6.0 software (Mestrelab Research, S.L, Santiago de Compostela, Spain) and two-dimensional NMR spectra by CARA program (Version 1.8.4) freeware at (http://cara.nmr.ch/doku.php/home, accessed on 5 May 2025). Proton resonances were sequentially assigned according to the Wuthrich standard method [31] and are reported in Table S1 of the Supplementary Materials. NMR data are accessible by BMRB entry 35009. For the spin-label experiments, a few microliters of a stock solution of 16-doxylstearic acid (16-DSA) in DMSO-d_6_ was added to the DPC peptide samples to achieve a 1:1 ratio spin-label/micelle.

### 4.14. Structure Determinations

The NOE-based distance restraints were derived from NOESY spectra recorded with a mixing time of 300 ms. The NOE cross-peaks were integrated with CARA and converted into upper distance bounds using the CALIBA program [66] incorporated into CYANA [40]. Geminal protons were chosen as distance reference with 2.0 Å. Inter-proton NOE-derived distances were applied as upper bounds in the structure calculation performed by the CYANA simulated annealing protocol [40]. Starting from 100 randomized conformers, structures were generated using the standard CYANA simulated annealing protocol with 20,000 torsion angle dynamics steps per conformer. The statistical treatment of the structures obtained is reported in Table S1. MolMol [67] software (Version 6.0.2-5475) was used to analyze the structures and Chimera-UCSF [40] to cluster them by similarity. Chosen three-dimensional models were visualized by PyMOL software (Version 3.0) (DeLano, CA, USA (2002)).

### 4.15. Cell Culture

Vero cells (code BCRT 0245), supplied by the Rio de Janeiro Cell Bank (Rio de Janiero, Brazil), were cultured and subcultured weekly using 0.25% trypsin-EDTA solution once they reached 80–100% confluence. After dissociation, the cells were washed and resuspended in RPMI 1640 medium supplemented with 10% fetal bovine serum (FBS). These cultures were then maintained at 37 °C in a humidified incubator with 5% CO_2_. The cultures were used for both screening assays and propagation of cultured *T. cruzi* trypomastigotes.

### 4.16. Trypanosoma cruzi

*T. cruzi* clone Dm28c (TcI), genetically modified to express luciferase (Dm28c-Luc), was kindly provided by Dr. Cristina Henriques (Oswaldo Cruz Institute—Fiocruz) [68]. The parasites were cultured in Vero cells maintained in RPMI 1640 medium supplemented with 10% FBS. At four days post-infection (dpi), the supernatant containing trypomastigotes was harvested, and the parasites were centrifuged at 800× *g* for 20 min at 4 °C. The resulting parasites were counted using a Neubauer chamber and subsequently used for phenotypic screening and studies of therapeutic combinations.

### 4.17. Cytotoxicity Assay

Vero cells were seeded at a density of 1.5 × 10^4^ cells/well in white 96-well plates with clear bottoms. After 24 h of culture, the cells were treated with increasing concentrations of Temporizin-1 or benznidazole (Bz), ranging from 3.12 to 100 µM for Temporizin-1 (3.12, 6.25, 12.5, 25, 50, and 100 µM) and 15.62 to 500 µM (15.62, 31.25, 62.5, 125, and 500 µM) for benznidazole. After an incubation period of 72 h, cell viability was assessed using CellTiter Glo reagent (Promega, Milan, Italy), which induces cell lysis and generates luminescence proportional to the intracellular ATP level. Luminescence was measured using a Glomax reader (Promega), and CC_50_ values were calculated by linear regression analysis using GraphPad Prism software (version 8.2.1). A low concentration of dimethyl sulfoxide (DMSO) (<1%) was employed as a negative control. Results are expressed as the means ± standard deviations of at least three independent experiments, each performed in duplicate.

### 4.18. Anti-T. cruzi In Vitro Assay

The susceptibility of *T. cruzi* (Dm28c-Luc) to treatment with Temporizin-1 was evaluated in both trypomastigote and amastigote stages. For the trypomastigote assays, 1 × 10^6^ parasites were inoculated into 96-well white plates with clear bottoms. After a 24 h exposure to different concentrations (0.39, 0.78, 1.56, 3.12, 6.25, 12.5, 25, and 50 µM) of the Temporizin-1 or Bz, D-luciferin (300 µg/mL) was added to measure luciferase activity, serving as a viability indicator for the parasites. Luminescence was measured using the Glomax reader (Promega). To assess the effect of Temporizin-1 on intracellular amastigotes, Vero cells (1.5 × 10^4^ cells/well) were cultured and then infected with *T. cruzi* Dm28c-Luc at a 10:1 parasite-to-host cell ratio for 24 h. Following infection, the cultures were treated with serial dilutions of the compounds (0.39, 0.78, 1.56, 3.12, 6.25, 12.5, 25, and 50 µM) for 72 h at 37 °C. The viability of the parasite was then measured by luminescence after the addition of D-luciferin (300 µg/mL). The IC_50_ values, representing the concentrations required to achieve a 50% reduction in viable parasite population, were calculated using linear regression analysis with GraphPad Prism software version 8.2.1. The selectivity index (SI) was derived from the ratio of the CC_50_ in Vero cells to the IC_50_ against *T. cruzi*, providing insight into the therapeutic margin of the tested compounds. DMSO concentrations (<1%) served as a negative control. Each experimental condition was replicated in duplicate in a minimum of three independent trials [69].

### 4.19. Combination Assay

To investigate the interaction between Temporizin-1 and Bz, we used the isobologram approach in *T. cruzi* trypomastigotes [70]. We assessed combinations of varying concentrations of Bz and Temporizin-1 at ratios of 5:0, 4:1, 3:2, 2:3, 1:4, and 0:5, by adding these combinations to 96-well plates containing trypomastigotes (1 × 10^6^ parasites per well) for a 24 h treatment period. Following exposure, we evaluated parasite viability using luminescence measured by a Glomax reader after the addition of luciferin. For each treatment combination, we calculated the IC_50_ values and derived the fractional inhibitory concentration index (FICI) using the following equation: FICI = IC_50_ of the combination/IC_50_ of the individual compound. The mean FICI (×ΣFICI) represented the average across the combination groups. Interactions classification was based on ×ΣFICI values as follows: synergistic (×ΣFICI ≤ 0.5), additive (0.5 < ×ΣFICI < 4), or antagonistic (×ΣFICI > 4). Isobologram plots were generated using GraphPad Prism version 8.2.1, with mean FICIs derived from three independent experiments.

### 4.20. Membrane Permeability Assay

Cytoplasmic membrane damage was determined using steady-state fluorescence, as described before [71], with some modifications. Briefly, tissue cultured with trypomastigotes (1 × 10^6^) was treated with 10 µM of Temporizin-1 for 24 h. Next, cultures were incubated with 10 µM of propidium iodide (PI) at 37 °C for 15 min in the dark. In addition, parasite membranes were stained with Concanavalin A-FITC (50 µg/mL) to allow visualization of glycoproteins on the parasite membrane. Cells were collected by centrifugation (10,000× *g* for 5 min) and washed three times in PBS. A final cell suspension was smeared onto a glass slide for imaging on an Axio Imager M2 microscope (Carl Zeiss, Oberkochen, Germany).

## 5. Conclusions

We demonstrated that the membranolytic activity of Temporizin-1 is driven by its intrinsic physicochemical features, including the net positive charge, amphiphicity, and hydrophobicity. These features promote strong interactions with lipid membranes, leading to pore formation and subsequent membrane disruption. Membrane damage was confirmed in trypomastigotes, and Temporizin-1 presents an increased selectivity against amastigotes. Combination therapy with benznidazole showed an additive effect, supporting further optimization of Temporizin-1 to increase selectivity against *T. cruzi*. In conclusion, by elucidating the relationship between structural modifications and biological activity, this study highlights key functional features that can be exploited to enhance selectivity and potency by applying chemical strategies and conjugation to a delivery system. Our results provide valuable insights into the molecular mechanisms underlying Temporizin-1 activity, offering important clues for the rational design of next-generation antimicrobials.

## Figures and Tables

**Table 1:** Biological activity of peptide Temporizin-1 against *Trypanosoma cruzi*.

Compounds	Anti-*T. cruzi* Activity (Mean ± SD)				Cytotoxicity (Mean ± SD)
	Trypomastigotes		Intracellular Amastigotes		
	IC_50_ (µM)	SI	IC_50_ (µM)	SI	CC_50_ (µM)
**Temporizin-1**	7.2 ± 0.2	3.8	1.9 ± 0.4	15.5	27.1 ± 1.9
**Bz**	18.6 ± 1.5	>26.8	2.4 ± 1.2	>208.3	>500

The mean values for IC_50_ and CC_50_ derived from three independent experiments and presented as mean ± standard deviation (SD); IC_50_ indicates the concentration inhabiting 50% of parasite viability; CC_50_ reports the concentration reducing Vero cell viability by 50%; the selectivity index (SI) = CC_50_/IC_50._

## Data Availability

All data are contained within the article and Supplementary Materials. The NMR structures of Temporizin-1 in H_2_O/D_2_O 90/10 are available in the Protein Data Bank with PDB code 9S5G and proton chemical shifts with BMRB ID 35009.

## Supplementary Materials

The following supporting information can be downloaded at: https://www.mdpi.com/article/10.3390/antibiotics14090913/s1, Figure S1: Residual percentage of the intensities (I) of NH-αCH cross peaks (I16-DSA/I0) measured in two TOCSY spectra of Temporizin-1 in DPC 50 mM acquired with (I16-DSA) and without (I0) 16-DSA; Figure S2: Chromatogram of Temporizin-1 obtained by an analytical HPLC (Jasco LC-NetII/ADC) equipped with Phenomenex Kinetex C18 column (5 µm, 100 Å, 150 × 21.2 mm) [linear gradient 10–90% MeCN (0.1% TFA) in H_2_O (0.1% TFA) over 20 min, flow rate of 1 mL/min, and monitored by UV detection at 220 nm]; Figure S3: ESI-MS of Temporizin-1; Table S1: CYANA structural statistic of Temporizin-1 in H_2_O/D_2_O 90/10 pH 5.

## References

[1] Hochberg N.S., Montgomery S.P. (2023). Chagas Disease. Ann. Intern. Med..

[2] de Sousa A.S., Vermeij D., Ramos A.N., Luquetti A.O. (2024). Chagas disease. Lancet.

[3] Pan American Health Organization (2019). Chagas Disease. https://www.paho.org/en/topics/chagas-disease.

[4] Schmunis G.A., Yadon Z.E. (2010). Chagas disease: A Latin American health problem becoming a world health problem. Acta Trop..

[5] Abras A., Ballart C., Fernandez-Arevalo A., Pinazo M.J., Gascon J., Munoz C., Gallego M. (2022). Worldwide Control and Management of Chagas Disease in a New Era of Globalization: A Close Look at Congenital *Trypanosoma cruzi* Infection. Clin. Microbiol. Rev..

[6] Nobrega A.A., Garcia M.H., Tatto E., Obara M.T., Costa E., Sobel J., Araujo W.N. (2009). Oral transmission of Chagas disease by consumption of acai palm fruit, Brazil. Emerg. Infect. Dis..

[7] Swett M.C., Rayes D.L., Campos S.V., Kumar R.N. (2024). Chagas Disease: Epidemiology, Diagnosis, and Treatment. Curr. Cardiol. Rep..

[8] World Health Organization (2023). Chagas Disease (Also Known as American Trypanosomiasis). https://www.who.int/news-room/fact-sheets/detail/chagas-disease-.

[9] Rassi A., Rassi A., Marin-Neto J.A. (2010). Chagas disease. Lancet.

[10] Nunes M.C.P., Beaton A., Acquatella H., Bern C., Bolger A.F., Echeverria L.E., Dutra W.O., Gascon J., Morillo C.A., Oliveira-Filho J. (2018). Chagas Cardiomyopathy: An Update of Current Clinical Knowledge and Management: A Scientific Statement From the American Heart Association. Circulation.

[11] Bern C., Messenger L.A., Whitman J.D., Maguire J.H. (2019). Chagas Disease in the United States: A Public Health Approach. Clin. Microbiol. Rev..

[12] Perez-Molina J.A., Crespillo-Andujar C., Bosch-Nicolau P., Molina I. (2021). Trypanocidal treatment of Chagas disease. Enferm. Infecc. Microbiol. Clin. (Engl. Ed.).

[13] Ochoa-Martinez P., Lopez-Monteon A., Lopez-Dominguez J., Manning-Cela R.G., Ramos-Ligonio A. (2025). Expression Analysis of Thirteen Genes in Response to Nifurtimox and Benznidazole in Mexican Isolates of *Trypanosoma cruzi* by Digital PCR. Acta Parasitol..

[14] Robledo S.M., Perez-Silanes S., Fernandez-Rubio C., Poveda A., Monzote L., Gonzalez V.M., Alonso-Collado P., Carrion J. (2023). Neglected Zoonotic Diseases: Advances in the Development of Cell-Penetrating and Antimicrobial Peptides against Leishmaniosis and Chagas Disease. Pathogens.

[15] Bucataru C., Ciobanasu C. (2024). Antimicrobial peptides: Opportunities and challenges in overcoming resistance. Microbiol. Res..

[16] Mookherjee N., Anderson M.A., Haagsman H.P., Davidson D.J. (2020). Antimicrobial host defence peptides: Functions and clinical potential. Nat. Rev. Drug. Discov..

[17] Ji S., An F., Zhang T., Lou M., Guo J., Liu K., Zhu Y., Wu J., Wu R. (2024). Antimicrobial peptides: An alternative to traditional antibiotics. Eur. J. Med. Chem..

[18] Nicoletti R., Bellavita R., Falanga A. (2023). The Outstanding Chemodiversity of Marine-Derived Talaromyces. Biomolecules.

[19] Hagemann C.L., Macedo A.J., Tasca T. (2024). Therapeutic potential of antimicrobial peptides against pathogenic protozoa. Parasitol. Res..

[20] Adade C.M., Oliveira I.R., Pais J.A., Souto-Padron T. (2013). Melittin peptide kills *Trypanosoma cruzi* parasites by inducing different cell death pathways. Toxicon.

[21] Osorio-Mendez J.F., Pardo-Rodriguez D., Rocha-Roa C., Toro L.J., Munoz-Tabares L., Recalde-Reyes D.P., Cala M.P. (2025). Exploring the mechanisms of action of the antimicrobial peptide CZS-5 against *Trypanosoma cruzi* epimastigotes: Insights from metabolomics and molecular dynamics. Parasites Vectors.

[22] Rojas-Pirela M., Kemmerling U., Quinones W., Michels P.A.M., Rojas V. (2023). Antimicrobial Peptides (AMPs): Potential Therapeutic Strategy against Trypanosomiases?. Biomolecules.

[23] Berhe H., Kumar Cinthakunta Sridhar M., Zerihun M., Qvit N. (2024). The Potential Use of Peptides in the Fight against Chagas Disease and Leishmaniasis. Pharmaceutics.

[24] Madison M.N., Kleshchenko Y.Y., Nde P.N., Simmons K.J., Lima M.F., Villalta F. (2007). Human defensin alpha-1 causes *Trypanosoma cruzi* membrane pore formation and induces DNA fragmentation, which leads to trypanosome destruction. Infect. Immun..

[25] Souza A.L., Faria R.X., Calabrese K.S., Hardoim D.J., Taniwaki N., Alves L.A., De Simone S.G. (2016). Temporizin and Temporizin-1 Peptides as Novel Candidates for Eliminating *Trypanosoma cruzi*. PLoS ONE.

[26] Edwards I.A., Elliott A.G., Kavanagh A.M., Zuegg J., Blaskovich M.A., Cooper M.A. (2016). Contribution of Amphipathicity and Hydrophobicity to the Antimicrobial Activity and Cytotoxicity of beta-Hairpin Peptides. ACS Infect. Dis..

[27] Dathe M., Wieprecht T., Nikolenko H., Handel L., Maloy W.L., MacDonald D.L., Beyermann M., Bienert M. (1997). Hydrophobicity, hydrophobic moment and angle subtended by charged residues modulate antibacterial and haemolytic activity of amphipathic helical peptides. FEBS Lett..

[28] Johnson T.S., Bourdine A.A., Deber C.M. (2023). Hydrophobic moment drives penetration of bacterial membranes by transmembrane peptides. J. Biol. Chem..

[29] Kyte J., Doolittle R.F. (1982). A simple method for displaying the hydropathic character of a protein. J. Mol. Biol..

[30] White S.H., Wimley W.C. (1998). Hydrophobic interactions of peptides with membrane interfaces. Biochim. Biophys. Acta.

[31] Wimley W.C., White S.H. (1996). Experimentally determined hydrophobicity scale for proteins at membrane interfaces. Nat. Struct. Biol..

[32] Smith T.K., Butikofer P. (2010). Lipid metabolism in *Trypanosoma brucei*. Mol. Biochem. Parasitol..

[33] Grieco P., Carotenuto A., Auriemma L., Saviello M.R., Campiglia P., Gomez-Monterrey I.M., Marcellini L., Luca V., Barra D., Novellino E. (2013). The effect of d-amino acid substitution on the selectivity of temporin L towards target cells: Identification of a potent anti-Candida peptide. Biochim. Biophys. Acta.

[34] LeVine H. (1993). Thioflavine T interaction with synthetic Alzheimer’s disease beta-amyloid peptides: Detection of amyloid aggregation in solution. Protein Sci..

[35] Parasassi T., De Stasio G., Ravagnan G., Rusch R.M., Gratton E. (1991). Quantitation of lipid phases in phospholipid vesicles by the generalized polarization of Laurdan fluorescence. Biophys. J..

[36] Manzo G., Carboni M., Rinaldi A.C., Casu M., Scorciapino M.A. (2013). Characterization of sodium dodecylsulphate and dodecylphosphocholine mixed micelles through NMR and dynamic light scattering. Magn. Reson. Chem..

[37] Sani M.A., Rajput S., Keizer D.W., Separovic F. (2024). NMR techniques for investigating antimicrobial peptides in model membranes and bacterial cells. Methods.

[38] Wishart D.S., Sykes B.D., Richards F.M. (1991). Simple techniques for the quantification of protein secondary structure by 1H NMR spectroscopy. FEBS Lett..

[39] Wishart D.S., Sykes B.D., Richards F.M. (1991). Relationship between nuclear magnetic resonance chemical shift and protein secondary structure. J. Mol. Biol..

[40] Guntert P. (2004). Automated NMR structure calculation with CYANA. Methods Mol. Biol..

[41] Pettersen E.F., Goddard T.D., Huang C.C., Couch G.S., Greenblatt D.M., Meng E.C., Ferrin T.E. (2004). UCSF Chimera-a visualization system for exploratory research and analysis. J. Comput. Chem..

[42] Brown L.R., Bosch C., Wuthrich K. (1981). Location and orientation relative to the micelle surface for glucagon in mixed micelles with dodecylphosphocholine: EPR and NMR studies. Biochim. Biophys. Acta.

[43] Pirtskhalava M., Vishnepolsky B., Grigolava M., Managadze G. (2021). Physicochemical Features and Peculiarities of Interaction of AMP with the Membrane. Pharmaceuticals.

[44] Stone T.A., Cole G.B., Ravamehr-Lake D., Nguyen H.Q., Khan F., Sharpe S., Deber C.M. (2019). Positive Charge Patterning and Hydrophobicity of Membrane-Active Antimicrobial Peptides as Determinants of Activity, Toxicity, and Pharmacokinetic Stability. J. Med. Chem..

[45] White S.H., Wimley W.C. (1999). Membrane protein folding and stability: Physical principles. Annu. Rev. Biophys. Biomol. Struct..

[46] Chan D.I., Prenner E.J., Vogel H.J. (2006). Tryptophan- and arginine-rich antimicrobial peptides: Structures and mechanisms of action. Biochim. Biophys. Acta.

[47] Kumar P., Kizhakkedathu J.N., Straus S.K. (2018). Antimicrobial peptides: Diversity, mechanism of action and strategies to im-prove the activity and biocompatibility in vivo. Biomolecules.

[48] Erdem Büyükkiraz M., Kesmen Z. (2022). Antimicrobial peptides (AMPs): A promising class of antimicrobial compounds. J. Appl. Microbiol..

[49] Giménez-Andrés M., Čopič A., Antonny B. (2018). The Many Faces of Amphipathic Helices. Biomolecules.

[50] El-Dirany R., Shahrour H., Dirany Z., Abdel-Sater F., Gonzalez-Gaitano G., Brandenburg K., Martinez de Tejada G., Nguewa P.A. (2021). Activity of Anti-Microbial Peptides (AMPs) against Leishmania and Other Parasites: An Overview. Biomolecules.

[51] Porta E.O.J., Kalesh K., Steel P.G. (2023). Navigating drug repurposing for Chagas disease: Advances, challenges, and opportunities. Front. Pharmacol..

[52] Barra T., Falanga A., Bellavita R., Laforgia V., Prisco M., Galdiero S., Valiante S. (2022). gH625-liposomes deliver PACAP through a dynamic in vitro model of the blood-brain barrier. Front. Physiol..

[53] Bellavita R., Buommino E., Casciaro B., Merlino F., Cappiello F., Marigliano N., Saviano A., Maione F., Santangelo R., Mangoni M.L. (2022). Synthetic Amphipathic beta-Sheet Temporin-Derived Peptide with Dual Antibacterial and Anti-Inflammatory Activities. Antibiotics.

[54] Francois-Martin C., Pincet F. (2017). Actual fusion efficiency in the lipid mixing assay—Comparison between nanodiscs and liposomes. Sci. Rep..

[55] Vitiello G., Falanga A., Galdiero M., Marsh D., Galdiero S., D’Errico G. (2011). Lipid composition modulates the interaction of peptides deriving from herpes simplex virus type I glycoproteins B and H with biomembranes. Biochim. Biophys. Acta.

[56] Struck D.K., Hoekstra D., Pagano R.E. (1981). Use of resonance energy transfer to monitor membrane fusion. Biochemistry.

[57] Tallmadge D.H., Huebner J.S., Borkman R.F. (1989). Acrylamide quenching of tryptophan photochemistry and photophysics. Photochem. Photobiol..

[58] Bellavita R., Maione A., Braccia S., Sinoca M., Galdiero S., Galdiero E., Falanga A. (2023). Myxinidin-Derived Peptide against Biofilms Caused by Cystic Fibrosis Emerging Pathogens. Int. J. Mol. Sci..

[59] Ozyigit I.E., Karakus E., Pekcan O. (2016). The modifier effects of chymotrypsin and trypsin enzymes on fluorescence lifetime distribution of “N-(1-pyrenyl)maleimide-bovine serum albumin” complex. Spectrochim. Acta Part A Mol. Biomol. Spectrosc..

[60] Bellavita R., Braccia S., Imbo L.E., Grieco P., Galdiero S., D’Auria G., Falanga A., Falcigno L. (2024). Exploring Fe(III) coordination and membrane interaction of a siderophore-peptide conjugate: Enhancing synergistically the antimicrobial activity. J. Inorg. Biochem..

[61] Wallner J., Lhota G., Schosserer M., Vorauer-Uhl K. (2017). An approach for liposome immobilization using sterically stabilized micelles (SSMs) as a precursor for bio-layer interferometry-based interaction studies. Colloids. Surf. B Biointerfaces.

[62] Bellavita R., Falanga A., Merlino F., D’Auria G., Molfetta N., Saviano A., Maione F., Galdiero U., Catania M.R., Galdiero S. (2023). Unveiling the mechanism of action of acylated temporin L analogues against multidrug-resistant Candida albicans. J. Enzym. Inhib. Med. Chem..

[63] Hudson S.A., Ecroyd H., Kee T.W., Carver J.A. (2009). The thioflavin T fluorescence assay for amyloid fibril detection can be biased by the presence of exogenous compounds. FEBS J..

[64] Amaro M., Reina F., Hof M., Eggeling C., Sezgin E. (2017). Laurdan and Di-4-ANEPPDHQ probe different properties of the membrane. J. Phys. D Appl. Phys..

[65] Yousif A.M., Ingangi V., Merlino F., Brancaccio D., Minopoli M., Bellavita R., Novellino E., Carriero M.V., Carotenuto A., Grieco P. (2018). Urokinase receptor derived peptides as potent inhibitors of the formyl peptide receptor type 1-triggered cell migration. Eur. J. Med. Chem..

[66] Guntert P., Braun W., Wuthrich K. (1991). Efficient computation of three-dimensional protein structures in solution from nuclear magnetic resonance data using the program DIANA and the supporting programs CALIBA, HABAS and GLOMSA. J. Mol. Biol..

[67] Koradi R., Billeter M., Wuthrich K. (1996). MOLMOL: A program for display and analysis of macromolecular structures. J. Mol. Graph..

[68] Henriques C., Henriques-Pons A., Meuser-Batista M., Ribeiro A.S., de Souza W. (2014). In vivo imaging of mice infected with bioluminescent *Trypanosoma cruzi* unveils novel sites of infection. Parasites Vectors.

[69] de Oliveira E.C., Lara L.D.S., Orlando L.M.R., Lanera S.D.C., de Souza T.P., Figueiredo N.D.S., Paes V.B., Mazzochi A.C., Fernandes P.H.M., Dos Santos M.S. (2025). Anti-Trypanosoma cruzi potential of new pyrazole-imidazoline derivatives. Molecules.

[70] Fivelman Q.L., Adagu I.S., Warhurst D.C. (2004). Modified fixed-ratio isobologram method for studying in vitro interactions between atovaquone and proguanil or dihydroartemisinin against drug-resistant strains of *Plasmodium falciparum*. Antimicrob. Agents Chemother..

[71] Rangel K., Cabral F.O., Lechuga G.C., Carvalho J.P.R.S., Villas-Bôas M.H.S., Midlej V., De-Simone S.G. (2021). Detrimental Effect of Ozone on Pathogenic Bacteria. Microorganisms.

