# Supplementary material for: Temporizin-1 Meets the Membranes: Probing Membrane Inser-Tion and Disruption Mechanisms
Source: Antibiotics (Basel). 2025 Sep 10;14(9):913. doi: 10.3390/antibiotics14090913 (PMC12466833; doi:10.3390/antibiotics14090913)
Supplement: Supplementary file 1 [file antibiotics-14-00913-s001.zip › antibiotics-3825047-supplementary.pdf]

Temporizin-1 Meets the Membranes: Probing Membrane Inser-Tion and Disruption Mechanisms

Rosa Bellavita [1,†], Sara Palladino [1,†], Karyne Rangel [2,3], Guilherme Curty Lechuga [2,3,4], Lorenzo Emiliano Imbò [5], Lucia Falcigno [1], Gabriella D'Auria [1], Leonardo da Silva Lara [4], Mirian Cláudia de Souza Pereira [4], Salvatore Giovanni De-Simone [2,3], Stefania Galdiero [1,*] and Annarita Falanga [5,*]

[1] Department of Pharmacy, School of Medicine, University of Naples Federico II, Via Domenico Montesano 49, 80131 Napoli, Italy; rosa.bellavita@unina.it (R.B.); sara.palladino@unina.it (S.P.); falcigno@unina.it (L.F.); gabriella.dauria@unina.it (G.D.)
[2] Center for Technological Development in Health (CDTS), National Institute of Science and Technology for Innovation in Neglected Population Diseases (INCT-IDPN), Oswaldo Cruz Foundation (FIOCRUZ), Rio de Janeiro 21040-900, Brazil; karyne.rangelk@gmail.com (K.R.); guilherme.curty@fiocruz.br (G.C.L.); salvatore.simone@fiocruz.br (S.G.D.-S.)
[3] Laboratory of Epidemiology and Molecular Systematics (LESM), Oswaldo Cruz Institute, Oswaldo Cruz Foundation (FIOCRUZ), Rio de Janeiro 21040-900, Brazil
[4] Cellular Ultrastructure Laboratory, Oswaldo Cruz Institute, Oswaldo Cruz Foundation (FIOCRUZ), Rio de Janeiro 21040-900, Brazil; leonardosilva.lara@hotmail.com (L.d.S.L.); mirian@ioc.fiocruz.br (M.C.d.S.P.)
[5] Department of Agricultural Sciences, University of Naples Federico II, Via Università 100, 80055 Portici, Italy; lorenzoemiliano.imbo@unina.it
* Correspondence: sgaldier@unina.it (S.G.); annarita.falanga@unina.it (A.F.)
† These authors contributed equally to this work.

Table of content

1. CYANA structural statistic of Temporizin-1 (Table S1) 2

2. A plot of the residual percentage of the intensities of NH-αCH cross peaks measured in two TOCSY spectra (Figure S1) 3

3. HPLC and ESI-MS spectra of Temporizin-1 (Figure S2-S3) 3-4

Table S1. CYANA structural statistic of Temporizin-1 in H_2O/D_2O 90/10 pH 5

distance restraints	194
intraresidue	161
sequential (|i - j| = 1)	26
medium-range (1< |i - j| ≤ 4)	7
**Violation statistics (100 structures)**	
CYANA TF ($Å^2$)	0.68 ± 0.92
**Residual Distance Constraint Violations (Å) for 100 str.**	
number > 0.2 Å	0
mean global backbone RMSD (Å)	1.49 ± 0.75
mean global heavy atom RMSD (Å)	2.43 ± 0.95
**Violation statistics (40 structures)**	
CYANA TF ($Å^2$)	0.25 ± 0.07
**Residual Distance Constraint Violations (Å) for 40 str.**	
number > 0.2 Å	0
mean global backbone RMSD (Å)	0.99 ± 0.26
mean global heavy atom RMSD (Å)	1.75 ± 0.33
**Violation statistics (100 structures)**	2.04 ± 0.45

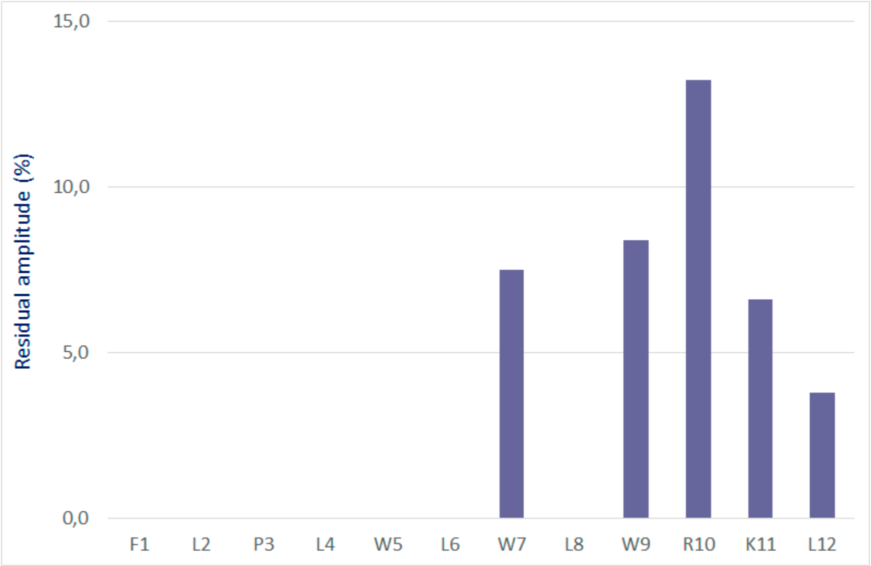

Figure S1. Residual percentage of the intensities (I) of NH-αCH cross peaks (I16-DSA/I0) measured in two TOCSY spectra of Temporizin-1 in DPC 50 mM acquired with (I16-DSA) and without (I0) 16-DSA.

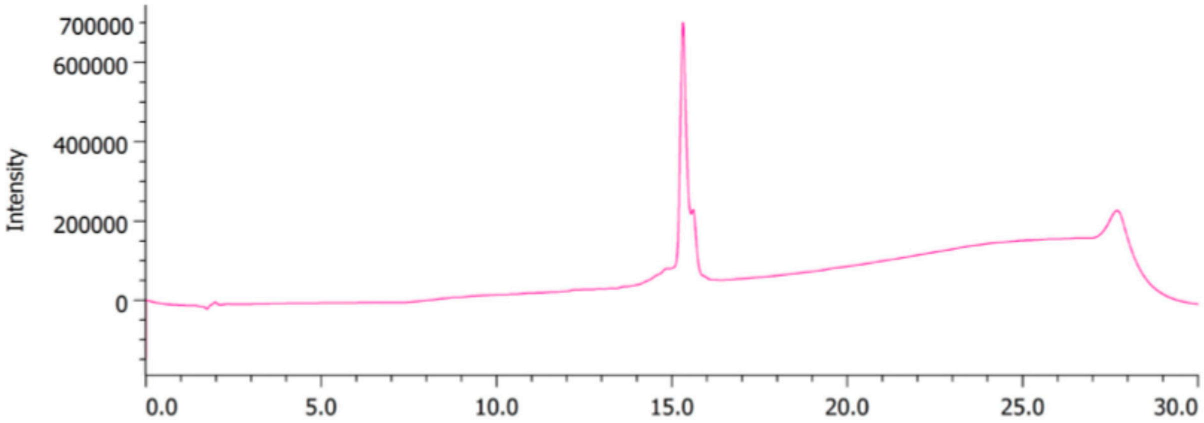

Figure S2. Chromatogram of Temporizin-1 obtained by an analytical HPLC (Jasco LC-NetII/ADC) equipped with Phenomenex Kinetex C18 column (5 µm, 100 Å, 150 × 21.2 mm) [linear gradient 10-90% MeCN (0.1% TFA) in H_2O (0.1% TFA) over 20 min, flow rate of 1 mL/min, and monitored by UV detection at 220 nm.

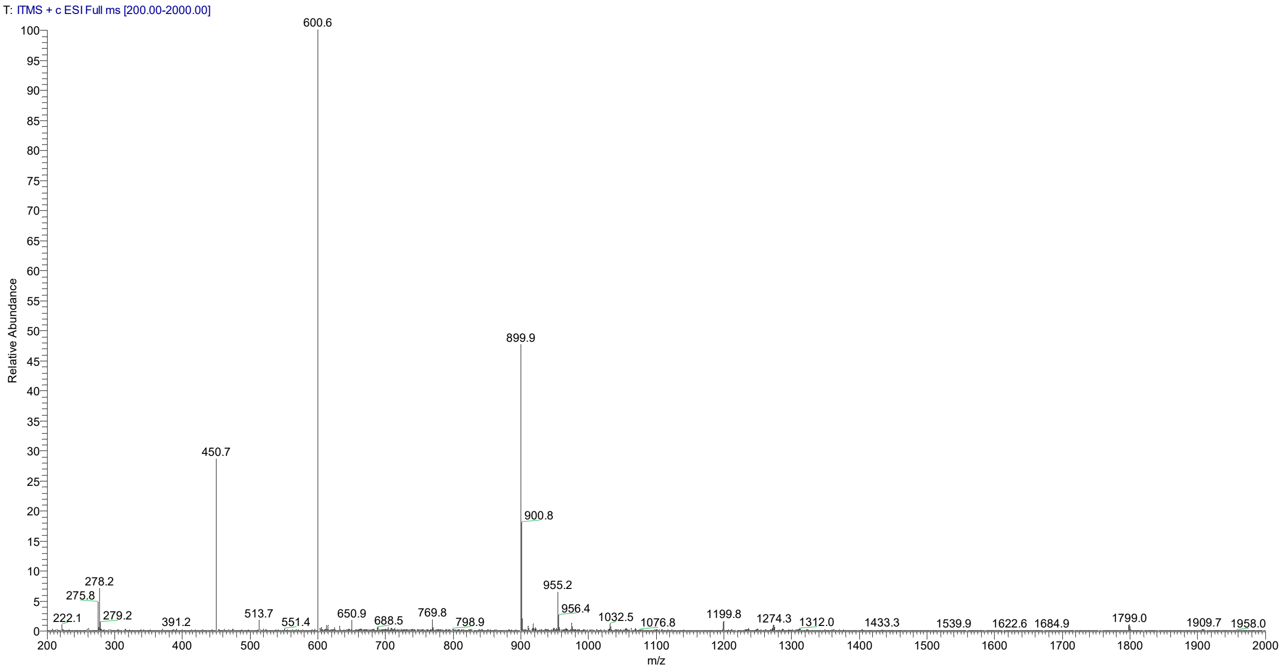

Figure S3. ESI-MS of Temporizin-1. Calculated mass: $[M+2H]^+/2=900.0$; $[M+3H]^+/3=600.3$; $[M+4H]^+/4=450.5$; Found mass: $[M+2H]^+/2=899.9$; $[M+3H]^+/3=600.6$; $[M+4H]^+/4=450.7$.
